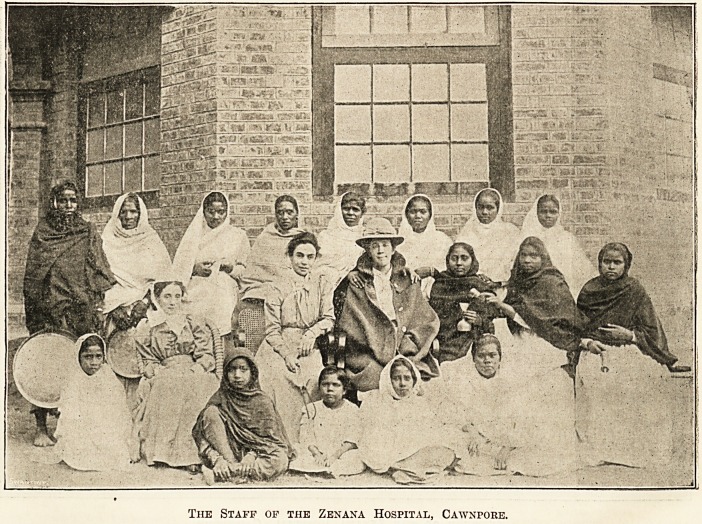# The Hospital. Nursing Section

**Published:** 1903-10-03

**Authors:** 


### The Hospital.

### Nursing Section.

Contributions for this Section of "The Hospital" should be addressed to the Editor, "The Hospital" Nursing Section, 28 & 29 Southampton Street, Strand, London, W.C.

No 888.—Vol. XXXV. *SATURDAY, OCTOBER* 3, 1903.

### Notes on News from the Nursing World.

### OUR CHRISTMAS DISTRIBUTION.

It is with much pleasure that we announce the fulfilment of the hope which we expressed on September 19th. We are able this week to acknowledge the receipt of the first contributions to our Christmas distribution of articles of clothing for the use of patients in hospitals and infirmaries. The arrival of two parcels from Miss Kate Saczkovie, Mitcheldean, No. 37 Royal National Pension Fund, and Miss E. Sharwood, Thames Ditton, thus early may be regarded as an indication of the still increasing interest which is taken by our readers in the distribution. Now that a start has been made we trust that large and small parcels will continue to come to us every week, addressed to the Editor, 28 & 29 Southampton Street, Strand, London, W.C., with "Clothing Distribution" written on the outside.

### THE HALF-TRAINED NURSE IN PARIS.

It will be recollected by some of our readers that in 1898 we published an exhaustive series of articles on the nursing question in Paris by Sir Edmund Spearman. The work of the gradual laïcisation of the hospitals in the French capital was described in detail from the time when the Paris Municipal School of Nursing was worked in 1878 until the time when every public hospital in Paris, with the exception of the Hotel Dieu—the mother-home of all nursing establishments—and the great Saint Louis Hospital were placed under the charge of lay matrons. In to-day's issue we give an account of the nursing as it is carried on under the auspices of the Paris Municipal School of Nursing. It will be seen that the condition of affairs is at present very far from satisfactory. Certificates, it is true, are insisted upon by the Municipality before the nurses can be promoted, but as these are often granted at the end of a single year, and can be earned either by a nurse in the wards, or a woman who studies in her own home and has never seen a patient, their value is obviously small, and, necessarily, may be a source of serious danger to the public. Contrasting this system with that which is described by our Commissioner as prevailing at the Hospital of St. John and St. Elizabeth in London, it must be acknowledged that the advantage lies with the nun who has received hospital training rather than with the half-trained but certificated French lay nurse.

### A NEW DEPARTURE AT GUY'S HOSPITAL.

A course of lectures for the benefit of past nurses is about to be given at Guy's Hospital, and it is hoped that this may become an annual fixture for the autumn term. The course, which is in connection with the Nurses' League of past and present nurses, is designed for the benefit of those who, having passed through the training school, and taken up private or other work which does not necessarily bring them into contact with new developments in medical and surgical nursing, antiseptics, etc., feel the need of keeping up to date in their work. The lectures, six in number, will be delivered by the four house surgeons, one house physician, and one obstetrical surgeon in the lecture rooms at the Nurses' Home. There will be a small subscription, and for the benefit of members who are unable to be present the lectures will be fully reported, typed, and sent to them by post. So greatly is the prospective course appreciated by the nurses that more than a hundred members of the League have already sent in their names. The first lecture will be given during the third week in October.

### KING'S COLLEGE HOSPITAL.

Although the technical year for the training of the nurses at King's College Hospital does not begin until November, we are able to state that the syllabus of lectures by members of the medical staff attached to the hospital will be precisely the same as that of last year, with certain improvements. One of these is, that with a view to preventing the heavy physical and mental strain on the nurses, the order in which the various subjects are taken will be different. For example, it has been customary to follow the course on physiology and medical nursing, for first year nurses, by one on anatomy and surgical nursing. This will now be placed last in order, the lectures on special subjects, *e.g.*, hygiene, the nursing of diseases of the eye, ear, and throat, following immediately on the physiology course. The same plan will be followed with regard to the second year's course, which runs simultaneously with the first, *i.e.*, the lectures on obstetric nursing and on the nursing of sick children will follow those on physiology and medical nursing, the course on anatomy and surgical nursing being placed last. The main courses consist of 10 lectures each, and are followed by classes held by the home sister. Some important additions to the practical and theoretical work are also to be made.

### ROYAL FREE HOSPITAL.

The autumn lectures to the nursing staff at the Royal Free Hospital have already begun. Part of the teaching consists of a course of lectures given by members of the medical and surgical staff duly appointed for the purpose, the lecturers being Dr. A. G. Phear, physician, and Mr. W. H. Evans, surgeon. The first lecture is given on Friday, this week, the subject being "Bones." It will also be treated on the two following Fridays, the 9th and 16th. The subject dealt with on the 23rd will be "Joints," and that on the 30th "Muscles." During November the subjects will be as follow: 6th, the "Alimentary Canal," etc.; 13th, "The Blood"; 20th, "The Circulation"; 27th, "Respiration"; while for three Fridays in December the subjects will be: 4th, "Digestion"; 11th, "The Kidneys and Skin, Heat of the Body"; 18th, "The Nervous System, Sight, and Hearing."

### ROYAL HOSPITAL FOR WOMEN AND CHILDREN.

Although it is anticipated that the roof of the new building of the Royal Hospital for Women and Children in Waterloo Bridge Road will be on in a very few weeks, the prospect of completing the scheme by the erection of suitable quarters for the nursing staff appears to be somewhat remote, only some £14,000 of the £50,000 required for the entire rebuilding having been so far received. The laying of the first stone of the new buildings by the Duchess of Albany on October 26th will, it is confidently expected, give an impetus to the fund, and this will enable the plans for the home in Stamford Street to be dealt with in detail. In the meantime the nursing staff will be accommodated on the top floor of the hospital. There will be in all 13 bedrooms, one of which, with separate bath-room, will be assigned to the matron. It is, of course, very desirable that the authorities should be in a position to proceed with the erection of the permanent home as soon as possible.

### THE NORTH-WESTERN POOR LAW CONFERENCE AND NURSING.

At the North-Western Poor Law Conference in Blackpool last week the subject of workhouse management was discussed, and Mr. H. Meadows, of the Bucklow Union, dwelt on the need of exercising care in the selection of nurses for the infirmaries. We agree with him that kindness of heart and gentle treatment are a *sine quâ non* in the treatment of the old and infirm, but his remark that, "in the case of a nurse, character should stand before a diploma" is open to misconception. The importance of character cannot be exaggerated, but even character without a certificate of capacity is not enough. Dr. Rhodes said that something more than either of these was needed as regarded nurses, and "that was a sufficiency of them." But nothing less than a sufficiency of nurses whose capacity and character are alike unimpeachable will ensure adequate attention on the sick in Poor Law infirmaries. To bring this about it is essential to relieve the superintendent nurse from any responsibility to the untrained matron, and it is satisfactory that at any rate one speaker at the conference insisted upon this as a vital point in the reform of the system.

### CENTRAL MIDWIVES BOARD.

A meeting of the Central Midwives Board was held on Thursday last week. Dr. Champneys was re-elected chairman until the first meeting in April, 1904. A letter was read from the Clerk of the Council enclosing a sealed copy of the rules as approved by the Privy Council in August, 1903. The secretary reported that the rules and forms were now printed and ready for publication and were on sale at Messrs. Spottiswoode and Co., Ltd., 54 Gracechurch Street, E.C., the rules at 6d. and 8d., and the forms 1d. each. The Secretary was instructed to complete the registration of the rules and forms, so as to preserve the copyright. A letter was read from the Hon. Sec. of the Manchester Southern and Maternity Hospital, enclosing an application for recognition as a training school for midwives under the regulation of the Board; the further consideration of this matter was adjourned, and the Secretary was instructed to ascertain whether the application of the hospital included a request for the recognition of their certificate as a sufficient qualification under the Act. Applications were read from registered medical probationers for recognition as teachers under section 6 of the rules, and also several applications from ladies desiring to be trained as midwives at a nominal fee. As to the latter, the secretary was instructed to refer any such applicants to the Association for Promoting the Training and Supply of Midwives, 12 Buckingham Street, Strand, where all information relative to the training and work of midwives might be obtained.

### CONFERENCE OF AMERICAN SUPERINTENDENTS OF TRAINING SCHOOLS.

On Wednesday, Thursday, and Friday, October 14th, 15th, and 16th, the American Society of Superintendents of Training Schools will meet in conference at Pittsburg. After the preliminary proceedings, which are to include addresses of welcome, the President, Miss Ida F. Giles, Superintendent of Nurses at the Homœopathic Hospital, Pittsburg, will deliver her address. The principal subjects introduced will be "The Power and Responsibility of the Society in Public Action," by Miss L. L. Dock; "The Better Teaching of Hygiene in Training Schools for Nurses," by Miss McIsaac; and "Some Common Weaknesses in Hospital Construction," by Miss A. M. Goodrich. Brief papers embodying suggestions as to methods of bringing the "Study of Current Events" into the training school will also be read, and one afternoon will be devoted to the description and demonstration of new nursing methods and appliances.

### NURSES AND PUBLIC WORSHIP.

Exception has been taken by certain bodies in Kirkcaldy to the presence at church of two of the nurses attached to the Kirkcaldy Infectious Diseases Hospital. It is not mentioned whether the nurses were recognised because of their uniform, or were known personally to the ladies who objected to them. If nurses engaged in attending cases of infectious diseases do not take stringent precautions against carrying infection they are always a source of danger when they go among the public, whether they are in uniform or not. But if they do they are no more likely to be the channel of contagion than doctors. We may add that if the presence of nurses at church is to be resented because they smell of disinfectants, as appears from the statement of a correspondent to have been the case in another part of Scotland, it would seem that their efforts to protect the public sometimes constitute a grievance.

### YORK NURSES IN BOARDED-OFF CRIBS.

At a meeting of the York Board of Guardians last week, the report of a sub-committee dealing with the question of accommodation for the workhouse nurses was considered. It was stated in the report that the nurses' rooms were most unsuitable, and that if quarters were built for them on adjoining ground there would be more room available for hospital needs and for the accommodation of inmates.

One of the Guardians maintained that the proposed work would cost something like £3,000. He knew that it had often been spoken of as needful, but other Guardians wanted, in addition, tennis and croquet grounds "and such like nonsense." What was undoubtedly required was a tramp ward, workshops, and other useful things, but not luxuries. He proposed that the part of the report relating to the nurses' quarters be deleted for the present. Another member of the board, in seconding the amendment, added that he did not think the Local Government Board would allow the nurses' quarters to be so far from the infirmary wards as was proposed. A Guardian, who demurred to this opinion, asserted that there was no fear of any difficulty in obtaining the sanction of the Local Government Board, and affirmed that "it was absolutely cruel to ask the nurses to pass their time in the present quarters, which consisted of boarded off cribs." Now that the question of building a nurses' home has at last been started, we hope that the York Guardians will see that the movement is carried through without delay.

### TO HELP NURSES IN THEIR WORK

The lady who in August offered through our columns to give a bicycle to a district nurse, now sends us the full particulars of the result. The number of applications rose, before she had finished receiving letters, to 60, and in consequence of her appeal in a Manchester paper, asking any ladies who had bicycles to spare, to communicate with her, she received several machines, while one correspondent, an invalid, was so much pleased with the idea of being able to help nurses in their work, that she sent the money for the purchase of two bicycles. Miss Greg states that she has done her best to help the most urgent cases. This, of course, is all that she could do ; but we hope that the publication of her letter will suggest to the mind of many more persons who possess discarded bicycles, or who can afford to emulate the example of the invalid lady, an admirable mode of doing a kindness whose effect is two-fold, since helping a nurse in her work means enabling her the better to tend the sick.

### GARDEN PARTY AT GLASGOW.

On Saturday week an "at home," in aid of the building fund, was held in the grounds of the Victoria Infirmary, Glasgow, a new wing being badly needed. The visitors were received by the matron, Miss Macfarlane. Refreshments were served out of doors. The assistant matron, night superintendent, and sisters assisted the lady governors at the tea-tables. The nurses, relieving one another in turn in the wards, also helped in entertaining the guests. They were all in uniform. The medical superintendent and staff took charge of the various amusements, which consisted of tennis, croquet, "Aunt Sally," etc. Several concerts were held in a large marquee which had been erected for the occasion. The 3rd L.R.Y. band played at intervals during the afternoon. Many of the guests took the opportunity of visiting the wards of the infirmary, and the nurses' home, which is practically new and very compact. The entertainment was a considerable financial success.

### THE QUESTION OF A SPECIALIST'S FEE.

The Local Government Board cannot be blamed for keeping a sharp look out on the expenditure of the Poor Law authorities, but the managers of the Poplar and Stepney Sick Asylum have given a conclusive answer to an inquiry respecting the payment of a fee of three guineas to a specialist who had been consulted by the medical superintendent during the illness of one of the nurses. The Local Government Board wanted to know whether the illness of the nurse was contracted by her while she was discharging her duties. The reply was that she undoubtedly contracted ophthalmia while she was looking after ophthalmic patients. If this had been indicated in connection with the item, we do not suppose that the charge would have been challenged. In the circumstances, the advice of a specialist was very properly sought.

### NIGHT DUTY AT NEWTON ABBOT.

At the last meeting of the Newton Abbot Guardians a nurse who had only been in the Guardians' service for three months tendered her resignation as she had obtained another post. Father Atkins, at the request of the Visiting Committee, raised the question of the nurses on night duty having the use of the duty-room instead of being obliged to sit in the wards when their presence there was not needed. He stated that the resolution of the Board, passed to that effect on July 19th, 1899, had been set aside and had been the cause of a great deal of the difficulty. The master, who was recently appointed, replied that he was unaware of the resolution, and that the nurses were entirely under the charge of the superintendent nurse. That is as it should be, and we trust that the superintendent nurse will consider it necessary for night nurses to remain in the wards during their hours of duty.

### SHORT ITEMS.

Following recent changes in the nursing staff at St. Bartholomew's Hospital, the entire scheme of instruction is undergoing revision. One alteration will be that the assistant matron, who has not hitherto taken part in the work of lecturing, will now be included in the list of lecturers.—The customary autumn course of lectures to the nursing staff of the Hospital for Sick Children, Great Ormond Street, is about to begin. The subject is medical and surgical nursing, and the lectures are given by the medical superintendent. Tutorial classes for juniors, held by the matron, follow each lecture. The usual examination of third year nurses who attended the lectures in the previous year will be held during October.—At the Hospital for Consumption, Brompton, lectures will shortly be resumed. These will be on anatomy and physiology, the lecturer being the resident medical officer.—The matron of the convalescent home at St. Leonards in connection with the Chelsea Hospital for Women, who recently resigned her appointment after holding it for 12 years, has asked to be allowed to retain the post for another year, and the council have acceded to her request.—Miss Marguerite E. Oakey, who has been superintendent nurse for the last two years at the Cheltenham Union Infirmary, is retiring from the nursing profession, and sails for India early in November. She has been the recipient of many presents and good wishes.—The new office for the matron and her staff at Guy's Hospital will be ready for occupation in about a fortnight. It is in a central position overlooking the square.

### Lectures on Ophthalmic Nursing.

By A. S. Cobbledick, M.D., B.S.Lond., Senior Clinical Assistant and late House-Surgeon and Registrar to the Royal Eye Hospital.

### LECTURE XX.—CATARACT.—VARIOUS TYPES, SYMPTOMS, DIAGNOSIS, AND TREATMENT.

*Cataract* is the term applied to any opacity in the lens, no matter what position or extent it may occupy.

As all rays of light have to pass through the lens to reach the retina, opacities in the lens impair vision in much the same manner as do opacities in the cornea, and the nearer the opacity is to the visual axis the more is the vision impaired. Cataracts may be variously classified.

1. According to age, viz., congenital, lamellar (in young people), senile.
2. According to the position in the lens, viz., nuclear, cortical (peripheral, anterior, posterior), anterior polar, posterior polar, zonular.
3. According to their consistence, viz , hard, soft.
4. According to the extent of the lens involved, viz., complete, partial.
5. According to causation, viz., senile, constitutional, *e g.*, as in diabetes, traumatic, glaucomatous.

Only those forms which are most commonly met with will be described in this course of lectures. The commonest is *senile cataract.* This form, as the title suggests, occurs in people of advanced age; the onset may not be noticed for some time, and the progress to maturity may be very slow, so that it is not uncommon to find the process beginning about the age of 50 or 55.

Amongst the earliest symptoms are inability to obtain good near vision with any glasses; double vision in the affected eye, *i.e.*, monocular diplopia, and dark specks, muscæ volitantes—moving in front of the eyes; note that these muscæ are only seen whilst the eyes are in motion, unlike those due to vitreous opacities which move about while the eye is at rest.

The majority of these cataracts begin in the cortical portion of the lens and at its periphery, so that ordinary inspection of the eye reveals very little defect, and it is not until the pupil has been enlarged by a mydriatic, *e.g.*, homatropine, so exposing that portion of the lens which lies behind the iris, that the defect becomes visible. There are two ways of inspecting the lens, viz., by shining an oblique light directly on to it by means of a convex lens, and by reflected light from the ophthalmoscopic mirror. In the former case, if striæ are present they appear as fine greyish lines on a black background, and in the latter case they appear as dark striæ on the red fundus reflex.

Fig. 1.

*i*, iris; *l*, lens with early peripheral striæ.

Fig. 2.
More advanced striæ.

As the peripheral striæ develop they become triangular with their bases at the periphery of the lens and their apices converging towards the centre. As the cataract ripens the interspaces between the striæ become opaque and gradually—sometimes quickly and oftentimes slowly—the whole lens becomes opaque so that fingers cannot be counted and nothing is left but a perception between light and darkness.

As the lens ripens it increases somewhat in size, so that it seems to be pressing the iris forwards; as a cataract is maturing it should be noticed that, with the oblique illumination, the iris throws *a shadow* on the portion of the cortex affected; as the cortex becomes more and more affected the *iris shadow* gets less and less; when there is no shadow it may be said that the cataract is mature. Most senile cataracts begin in the cortex and at the periphery; there is a form, however, beginning in the centre around the nucleus.

Fig. 3.

The result of this is that vision is impaired very early and is unimproved by glasses. Patients so afflicted always say they can see better in the dusk; this means that they see around the edge of the cataract, as the pupil dilates in a subdued light. This condition can be obtained permanently by using weak atropine drops (gr. i. ad. ℥i.).

For a similar reason patients with the opacities at the periphery of the lens see better in a strong light, which causes the iris to contract.

Fig. 4.

Normal lens. Lenticonus posticus.

in sagittal section.

Another not uncommon form of senile cataract is that in which patients become myopic through considerable change of shape of the posterior surface of the lens: this condition is termed *posterior lenticonus.*

Fig. 5.
As seen by reflected light. As seen in sagittal section.

Senile cataracts are hard on account of the density of the nucleus, which develops and grows in later life.

Lamellar or zonular cataracts are the next most common form: they occur in young children, and are always soft, as there is no nucleus.

Children with this form of bad sight have usually suffered

LECTURES ON OPHTHALMIC NURSING—*Continued.*

from rickets, bad feeding and convulsions, and the teeth show a deficiency of enamel.

The opacity takes the form of a ring: the centre of the lens is quite clear, then there is the opacity, and outside this the lens is again clear.

As a rule, this form of cataract remains stationary: it is practically always present in both eyes, and probably it is congenital, or begins in very early life. Attention is not drawn to the defect until the child's education is commenced.

*Traumatic Cataract* is not uncommon. Any perforating wound of the eyeball, in which the lens capsule has been penetrated, causes a cataract to form rapidly. This is due to the lens substance taking up water from the aqueous humour. The lens swells and becomes opaque in a few hours. A direct blow on the eye may also produce a cataract which rapidly matures, and apart from the history of injury and the age of the patient, cannot be distinguished from senile cataract.

Anterior and posterior polar cataracts are really situated without the lens system and are due, the former to corneal ulceration and the latter to disease in the vitreous.

Diabetic cataracts have much the same appearance as the senile forms. The rarer forms of cataract will not be dealt with in these lectures.

### Nursing by Sisters of Mercy.

INTERVIEW WITH THE MOTHER PRIORESS OF THE HOSPITAL OF ST. JOHN AND ST. ELIZABETH: BY OUR COMMISSIONER.

The question of nursing by nuns has been so prominent lately that an authoritative account of the system in force at the most important hospital in England which is under the care of a religious community will be read with interest. It is a little more than three years ago since the present building of the hospital of St. John and St. Elizabeth, which was founded in Great Ormond Street, London, in 1856, and removed in 1898 to Grove End Road, St. John's Wood, was opened. Those who attended the opening ceremony in July, 1900, will remember that it then appeared to possess every convenience and almost every modern appliance, but as Mr. Edward Blackett, one of the surgeons, told me the other day before my interview with the Mother Prioress, several important additions have been made. The ward for the open-air treatment for female patients suffering from consumption has been introduced, the ward for ordinary male patients has been opened, the children's ward and surgical ward have been painted and varnished, telephonic communication has been instituted, accommodation for paying patients has been provided, a resident medical salaried officer with his own quarters has been appointed, and by the addition of a consulting gynæcologist, a physician for diseases of the throat and ear, a gynæcologist, and an ophthalmic surgeon, the medical staff has practically been brought up to the level of a general hospital.

"So that," I said, when we had paid a visit to some of the wards, the beautiful church, and the remarkably fine and admirably equipped theatre, "you are now prepared to receive the full number of patients."

"If only," rejoined Mr. Blackett, "we had the means to open the whole of the beds in the male ward. There are now 85 beds opened, and we want to bring the number up to 102."

"What is your cost of maintenance?"

"It is one of the lowest in London, namely, £1 4s. a week. The turn-over of our patients is much greater than it was. In fact, it increases every year. We take in from 20 to 22 a month."

"Your desire is to rank as a general hospital?"

"Yes, though the cases are somewhat different. This is not, in any sense, a home for incurables, but we take in cases for long treatment. That is to say, they are not refused so long as there is any chance of doing good. But we do not admit hopeless cases like cancer and paralysis."

"And now as to the nursing?"

"I shall be very pleased to give you any information," replied the Mother Prioress. "As you know, the hospital is under the charge of myself and the Sisters of Mercy of the Convent of St. John, which is attached to the building."

"How many of these ladies are engaged in the wards under your direction?"

"About twenty-three. Ten are certificated."

### A Certificated Sister in Each Ward.

"Perhaps you would not mind naming some of the schools at which they received certificates?"

"The London Hospital, the Middlesex Hospital, the 'Dreadnought' Hospital at Greenwich, and the Leeds General Infirmary. One was for seven years sister at the London Hospital and was afterwards matron at another institution. All the ten have received full hospital training. We divide our nurses in just the same way as they are divided in a general hospital."

"I should like to have the details."

"Each ward contains 17 beds, and every ward sister is a certificated nurse. She has under her two who may not have been hospital trained, and a postulant who corresponds to a probationer in an ordinary hospital. These represent the nursing staff in the ward, and there is also a wardmaid in each ward."

### Day and Night Duty.

"What are the hours of duty?"

"The day is broken up into two parts. One set of nurses are on duty from half-past six in the morning until half-past one, and the other set from half-past one until half-past nine."

"With intervals for meals?"

"The former go out of the wards for breakfast and dinner, and the latter for tea and supper."

"I conclude that the night sisters come on duty at 9.30."

"Yes. There are two sisters on night duty in the hospital—one each side of the building. They remain on duty until 6.30 A.M. If we have any bad cases, which is not often, an extra nurse is put on duty for the night. The night sisters have supper before they go into the wards, and during the night they probably get a cup of tea in the ward kitchen."

"Are they required to wash the patients?"

"No, neither to wash the patients nor to make the beds. They simply attend to the wants of the patients during the night."

"The night sisters, I presume, are fully trained?"

"Always. Occasionally for night work we have lay sisters, but only if we are pushed. Of course we prefer that all the nurses should be members of the community. If we have lay nurses, they receive salaries. All the rest of the work is entirely voluntary."

"Am I right in assuming that the sisters do not have holidays?"

"Quite right. If they are unwell they have rest, and are sent away for a change; but holidays are not a part of our system."

"What is the period of probation in the convent?"

"Our members start as postulants, and become novices at the end of six months. At the end of two years they are professed and become sisters. During that period, and afterwards, they receive training in nursing. Up to the time they are professed they can leave whenever they like."

A Colleague of Florence Nightingale.

"And after they are professed?"

"It is always possible for a sister to go if her health breaks down, or if she comes to the conclusion that she cannot for any reason go on with the work. But such cases are very rare. On the other hand, we have sisters who have been here a great many years. Sister Anastasia has been with us more than fifty years. She nursed in the Crimea with Florence Nightingale. The superintendent has been here for 43 years."

"Has the superintendent charge of the nursing?"

"Her position corresponds very much to the matron of a general hospital."

Dress in the Wards.

"People sometimes suggest that the ordinary habit of a Sister of Mercy is unsuitable for nursing, but I saw in the theatre a sister who appeared to be wearing white linen."

"All the nurses practically wear white in the wards. Their black serge habits are covered with very large linen white aprons, and large over-sleeves. The head-dress also is white linen. The exception is when they are dusting, and then blue check aprons are worn."

"And the veils?"

"A postulant does not wear a veil. When she is in the theatre, she has nothing on her head. The novices wear a white veil in the wards, and the sisters a black one. They are pinned behind in order that they shall not be in the way."

A Ward in the Hospital of St. John and St. Elizabeth.

Training and Age.

"Is the theoretical training similar to that of other hospitals?"

"There are medical and surgical courses of lectures lasting three months in the winter and three months in the summer, the former by Dr. Harold, and the latter by Mr. Blackett. One day a week during the surgical course is devoted to practical bandaging. The surgical lectures have reference to the cases we take in, and if there is a specially interesting case it forms the subject of the lecture, the object being to describe the diseases which have to be dealt with. The lecturers have also to bear in mind the fact that the nurses are permanent, while not forgetting that new ones come in occasionally."

"Is there any examination?"

### NURSING BY SISTERS OF MERCY—*Continued.*

"No formal one, because there is no issue of a certificate, but the doctors ask questions and look over the notes made by the nurses. As to practice, the hospital-trained sister in each ward teaches the rudiments of nursing, sees that the beds are properly made, and that things generally are done as they should be."

"A rather important point is the age of admission."

"We take girls of 19 as postulants, but they are not put in charge of a ward until they are considerably older, and have gone through a course of theoretical and practical training."

### The Religious Question.

"Do you consider that the patients are affected by what I may call the religious influence?"

"The children are models in respect to behaviour, and I think that the adults are singularly patient. We ascribe this largely to the religious influence. At the same time I should like to emphasise the fact that the patients need not be Catholics."

"You mean that there is no regulation to that effect?"

"Quite so. The form of application for admission does not mention the question of creed, and no patient is interrogated on the subject. Some of the patients are Protestants, or Jews. We naturally require the lay nurses to be Catholics, as they are only engaged because our community cannot supply a sufficient number."

### Private Patients and Dispensers.

"Do the sisters nurse the private patients?"

"Not always; we have to obtain the services of lay nurses for them, if the wards are full. There are nine rooms for private patients who are under the care of their own physician or surgeon. The operation which was about to be performed when you were in the theatre was by an outside surgeon on a private patient. Our terms for private patients are from three guineas a week, and we could fill more rooms if we had them. We do not take in mental or infectious cases. The fees of the private patients are needed to help our income."

"By whom is your dispensing done?"

"We have three sisters who are qualified dispensers. Two qualified from the hospital, and one qualified before joining. They spend about a couple of hours a day in the dispensary."

"Lastly, as to your wants, on behalf of which, I believe, a special effort is to be made at an early date."

"Yes. Some kind friends with the sanction of the board are organising a fancy fair and fête to be held next May at the Empress Rooms, Royal Palace Hotel. We hope to get £5,000 to pay off our debt and enable us to open all our beds, but we could do with double."

### Hints on How to Start a District Nursing Association.

In these days when nearly every country town and village aspires to having its district nurse, a few hints on how to start a district nursing association may be found useful.

The object of a district nursing association is to nurse the sick poor in their own homes. By the word "poor" is not meant only those in actual poverty, but all working-class people and even others who are not able to afford the expense of a private nurse. District nursing consists of a daily round of visits paid by the nurse who goes from house to house doing what is necessary for each patient, and returning to her own rooms to have her meals, sleep, etc., making them her headquarters.

### The First Steps.

When the local promoters have agreed that it is desirable to have a district nurse, they should call a public meeting and invite specially the clergy and medical men and others likely to be interested to attend. The proposal should then be laid before the public and its object explained, and the feeling of the people probed with regard to starting an association. At this meeting it should be ascertained who are willing to stand as members of a General Committee. A few members should then be selected to act as a temporary Executive Committee. The temporary Executive Committee should meet privately at a convenient date to discuss matters and to transact business. Permanent executive members must be appointed, and rules drawn up, etc. If it be possible to get a trained nurse who has acted as county superintendent of district nurses to give advice and the benefit of her experience, it will be found a great help. One of the first steps in beginning an association is to secure the favour of the doctors, for unless they promise their help and support it will be very uphill work for the nurse; in fact it will be almost impossible for her to go on. A district nurse's work is so closely connected with that of the doctors' that she must work with them; she cannot very well nurse a patient if the medical man in attendance will not entrust her with his orders. Fortunately, however, most doctors welcome the advent of a district nurse and acknowledge what a great help she is to them.

### The Committee.

The committee can be enlarged to almost any extent, but experience has proved that it is best not to make it too large. It has also been found by experience that as a rule the wives of medical men are wise not to become members. The committee is composed of a General and an Executive Committee. The General Committee should consist of subscribers of 10s. and upwards. The clergy and medical men should be *ex officio* members or should be represented on the Executive Committee. The Executive Committee should comprise: (1) president, (2) treasurer, (3) secretary, (4) two or three other members. The secretary is generally the one who sees most of the nurse, and should, if possible, be someone within easy reach and possessed of tact and business capacity. If the district is made up of several scattered villages there might be a representative in each who would attend the committee meetings.

### Sources of Income.

There are various means of raising money to support a nursing association. It is probable that those chiefly interested in promoting the movement have already got promises of substantial support from wealthy people in the neighbourhood or even outside it and among their own friends, but all should be asked to subscribe, and those who are unwilling to become annual subscribers may be induced to give a donation, even if it be only a small one. In some places the poor are practically nursed free of charge—*i.e.* the services of the nurse are not refused to any of the poor and working classes who apply for them, though those who can afford to give a little are expected and encouraged to do so, but there are no fixed charges, all subscriptions and donations are voluntary. Yet the working classes, as a rule, can quite well afford to pay a small sum, and if it is judiciously managed it is a good plan to have fixed charges and to found the nursing association on the system of a benefit club. In some places a scale of fees is used, ranging from about 2s. up to 10s. per annum, according to the amount gained by the wage earner, as labourers, cottagers, farmers, artisans, tradesmen, etc. It is not altogether a satisfactory arrangement for it is sometimes rather a difficult and delicate matter dividing people into classes, and a labourer with one child is often better off than an artisan with a large family. Sometimes the minimum charge is fixed, and it is left to the people to give as much more as they can afford, *i.e.*, all the poor who give 2s. or upwards per annum are entitled to become members of the association and to the services of the nurse. Others who are not able to afford a private nurse may be willing to make a donation for the nurse's help. Boards of Guardians in many places give a yearly subscription, and it is only right they should do so, for the nurse very often attends to pauper patients who would otherwise have to go into the workhouse hospital. The very poor should be nursed free if their subscriptions are not paid for them. Endowed funds and trusts, which exist in some places for the benefit of the poor, may be used with advantage in helping to supply them with skilled nursing in times of sickness. If there are collieries or other large works in the district they often subscribe a substantial sum for the benefit of their workmen and their wives and children. Sometimes the workpeople agree to have ½d. per week kept back from their wages for the Nursing Association. There are many other ways to raise money for so worthy an object if only there are people enthusiastic enough to make the effort, such as:—1. Sales of work. 2. Jumble sales. 3. Concerts and other entertainments. 4. Special collections. 5. Proceeds of cricket matches, etc. 6. Garden parties.

Method of Collecting Money.

The district should be divided into parts and collectors appointed to each part. The collectors, who need not necessarily be members of the committee, should go round their several districts at regular times, whether it be monthly, quarterly, yearly, or so on, whichever is agreed upon. It is advisable to make a house-to-house visitation throughout the whole district, whatever the system of payment may be, at least once a year to try and secure new subscribers and collect any donations, not even despising 2d. or 3d. from people who will not become subscribers. People who subscribe an annual sum for the benefit of their poorer neighbours should be kind enough to send it to the collector of the district, and thus save her unnecessary trouble. There is a great art in collecting; some succeed in getting money out of people where others fail. To the poor ½d. per week may sound less than 2s. per annum, and it should be explained to them that 2s. per annum is really little more than ½d. per week. If non-subscribers apply for the nurse, they should be allowed to have her on condition that they pay extra. The nurse should never be expected to collect fees, but sometimes a patient who has probably just recovered, and is therefore in a grateful mood, offers her a fee that is due, or an extra donation; she should be allowed to take it and hand it on to the treasurer or collector, for if she does not take it, perhaps, by the time the collector goes round, the money is spent or the grateful mood gone.

*(To be continued.)*

### Sketches at a Children's Hospital.

### I.—The Royal Visit.

Great was the excitement throughout the hospital when the children were told that the Prince and Princess were going to visit them. We nurses were also in a flutter, Sister alone appeared unmoved. True she got out pinafores and fresh ribbons for the little girls, and put the boys into clean fresh suits, whilst Teddie looked irresistible in his muslin frock and openwork socks, with his golden hair curled by me. True she bought a few choice flowers, and arranged them about the ward as only she could arrange them. But she forbade us to teach the children anything. "Let them be natural," she said, "at any cost." We lifted all those who could be lifted up to the window to see the carriages arrive, for they came in State to please the children.

"Will she wear a crown?" asked Harry, the eldest.

"No," said Mary.

"She will!" cried Katie, red with indignation. "With precious jewels in it, too; like the hymn said we sung last Sunday."

"I don't believe it," said Harry, refusing to be convinced.

At last came the carriages, with their outriders and powdered flunkeys. One of these let down the steps of the carriage, and the Prince and Princess alighted, followed by one or two of their suite. They shook hands with the matron, and we saw them go inside.

"Who's that man in the blue coat and the white stockings?" asked Harry in an awed voice, pointing to the powdered servant. There was a pause, whilst all the little faces were pressed eagerly against the window panes. Then Katie, never to be beaten, said coolly, "Don't you know that? I saw at once it was the Queen's father." Her tone defied contradiction, and the children looked at her with more respect. We now hurried them back to bed, and got the ward tidy.

"Stay by Katie," said Sister in a whisper to me. "She is the only one I'm afraid of misbehaving."

We had a long wait before they came, and many were the amusing things said by the children; but I forget them now. I only remember how Sister enjoyed their speeches. At last the Royal visitors entered the ward, and to our surprise Harry bounded to meet the Princess, and clasping her hand in both his, said in an overjoyed tone, "O I'm so glad you have on a bonnet, for Katie said you would wear a crown with precious jewels in it."

"I have one at home," said the Princess, evidently amused. "But tell me where is Katie?"

"There she is," said Harry, pointing to the mischief of the ward. "She dare do anything; she cut the barber's hat into pieces."

The Princess laughed heartily, and with Harry still at her side made a tour of the cots, lingering with womanly pity at those that contained the small babies. I could not hear what she said until she got to the cot I was at, keeping guard over Katie.

"What toy would you like out of the hamper we have brought?" she asked.

"A gun," said Katie promptly, and without a trace of shyness.

The Prince looked at her with an indulgent smile. "What do you want with a gun?" he asked.

Katie flushed and looked at Sister.

"Speak, my dear, when the Prince questions you," said Sister.

"I want to fire at the barber," Katie confessed, and her eyes literally danced with mischief. "My, wouldn't he limp down the ward if I shotted him in his back!"

It was impossible not to smile, and the Prince laughed heartily, whilst I saw Harry give Katie an admiring glance for her bravery in speaking out.

"Why does she so dislike the barber?" asked the Prince of Sister.

But Katie answered for herself, in spite of my look and restraining touch on her arm. "Because he lets hair go in my eyes and tells me to hold my tongue, and because he takes snuff and makes me sneeze," she said, with rising indignation at the recollection of what she had suffered at his hands.

### SKETCHES AT A CHILDREN'S HOSPITAL—*Continued.*

The Prince turned away and twisted his moustache to hide a smile, and I heard him say to the doctor, "Is it true she cut his hat to pieces?"

And the doctor, with a laugh, confessed it was, adding that he had had to give him one to go home in. Teddie meantime had quite captivated one of the lady's hearts with his winning smile, and to my horror now insisted on leading her to see the sunroom. Getting decidedly mixed in his excitement, he finally, much to her amusement, led her into the bathroom, and then by way of apology said, "Ou can turn on the water, if ou like, me won't tell Sister, and it does splash."

I apologised, for at a look from Sister, I had followed them.

"Pray don't," said the lady sweetly. "These children are quite delightful, they are so natural. It is so much nicer for us when they have not been prepared for our visit."

I repeated this to Sister afterwards. She burst out laughing. "They certainly appeared to enjoy Katie," she said, but I was nervous I must confess, for she is such a terrible child, one never knows what to expect."

"Well, anyhow, Teddie was charming," I said, sticking up for my favourite.

"They were all charming," said Sister, in her nice way. "I am always proud of them on these occasions," and her face looked as if she meant it.

### II.—Punishment in the Ward.

In cot No. 20 there sat an old-fashioned looking boy whom I always said resembled an owl. He belonged to the very poorest class; he had been taught to lie, to steal, and to cheat. He was cunning, greedy, and silent, very pale and ill-looking, and curiously old for his years, and he was suffering from tubercular disease from which, sooner or later, he must die. He had not been very long in the ward before the children began to miss their money, small toys, and books. We also lost small things belonging to the ward, and it became only too evident that we had a thief in the ward.

Things were brought to a climax by the parents of the children complaining to Sister on visiting day. We were all sure it was James, yet as he was not allowed to get up, we could not conceive how he contrived to procure the things or secrete them. But one night when the night nurse was at her supper in the ward kitchen, when all the children, as she thought, were fast asleep, Dr. Sinclair, entering the ward in noiseless shoes, saw James creeping from locker to locker, looking like a little ghost in his long white nightgown and his bare feet. The Doctor glided behind a screen, determined to find out where the child hid his treasures. He saw James creep up to the sunroom, and he followed, and looking through the glass doors, saw the boy bury them in a deep hole in the wall just below the window; then looking round in a terrified way, as if half-conscious of being watched, he drew a toy stable before the hole and crept back to his cot. The Doctor meantime having made his escape, determined to let Sister Dale punish the culprit in her own wise way.

When Sister was told next morning she did not utter a word, but later I saw her lift James in a blanket and carry him into her own sitting-room. What she said to him she repeated to no one, but she put him back to bed looking very ashamed and miserable. That night when she went from cot to cot saying good-night, and pulling the clothes over the restless children, she passed Cot 20 without a glance. Next morning, when she spoke to each child as was her custom, she passed James without a word. All day her eyes never rested on him, she quietly ignored him. We began to be sorry for the boy. Some of us said it was too bad to keep a thing up against a child. One or two said a thrashing would have been kinder. All decided that Sister was for the first time in our recollection cruel. And we all tried to make up to him for her neglect, by being extra nice and attentive to him. But he would have none of our affection. He sat gloomy, miserable, and unhappy all day long, until at night he could bear it no longer. Sister was seated by the fire, sewing fresh lace on Jackie's dress. All the children were, we thought, asleep, and I was noiselessly putting out the clean linen for next morning, when of a sudden a piping little voice startled us.

"Sister," said James with a penitent sob, "I did steal the things, and I did hide them in the sunroom, you said you would love me if I told the truth, I have Sister. Oh, do speak to me!"

I turned swiftly to see what Sister would say or do. I saw her delicate face flush, as she went quickly to his cot. And then she lifted him in her arms and carried him to the fire. The ward was very silent, you could have heard a pin drop, so that I heard distinctly all she said.

"I am so pleased to find you a brave boy, who is not afraid to own to the truth," she said, and there was a glad ring in her voice. "And Jesus is glad, because no little boy who steals can go to His home, and He wants you there some day." And then she kissed his thin face tenderly. "When you want to steal again," she went on, "just call me, and I will try to help you to do right; will you, my boy?"

And little ragged James, the child of the gutter, put his thin arms round her neck, and kissed her beautiful face. "I will, Sister," he sobbed. "I never knew it were wrong afore, and I want to go to Jesus if you are going."

She put him back to bed then with tears in her eyes, and when he was asleep, his face had a happier expression than we had ever seen it wear. I said so to Sister, as we left the ward. She sighed. "Poor little thing," she said, "we have never seen him except ill and miserable; there is no telling how pretty he might grow, if we could teach him to trust us and be happy."

### III.—The Children at a Clinic.

The children in our ward never shrank from the students. On the contrary, they welcomed them as friends, for Sister, with her unerring tact, had contrived that it should be so.

"Very soon the students will be here," she would say aloud, early in the morning. "I am so glad they are so full of life and fun." And one of us nurses knew how to reply. "Yes, we should be dull without them."

"We shall miss them when holiday time comes." Sister would go on, speaking to us as she did her diet sheet. Then the students she also talked with. "Go and make friends with the children," she would say laughing. "They will tell you quite frankly about their pains, if you once get rid of their shyness."

And the students, shy as the children, would look at Sister whom they worshipped, and at her bidding follow her from cot to cot. Sister talking gaily without any effort, until after a little they found themselves joining in the fun, and unconsciously growing to know the children.

Presently, the visiting physician would enter to give his lecture in the ward. Both he and the house-surgeon, if dreaded, were loved, for Sister spoke of both as of angels who only existed to help little children in pain. "Dear kind doctor," she would say after he had gone. "How he does dislike hurting you little children."

"But he hurts me every day," some child would sob, with a keen remembrance of his dressing.

"I hurt you also," Sister would say, with a loving smile.

"But you all know I hurt you because I want to make you better, and send you home strong and well."

Of the house surgeon she spoke in a more familiar way. "If you take your medicine, Joe, I will tell Dr. S——," she would say, as if that was the honour of all others to be coveted, or she would see him coming along the corridor, and call cheerily to the children, "Here comes Dr. S—— to make us all laugh, run darlings to meet him, for this is his ward, and we must show him we are waiting for him." Such remarks as these robbed his visit of any dread, but I am wandering from my subject.

At 9.30 A.M., in walked the students. A curious crowd they were. Some rough raw lads from the country, some polished, the sons of gentlemen, some shy, some forward, some young, some old, but one and all fond of Sister. They used to greet her with a friendly smile each morning. One would say "Sister, would you dress my thumb? it's been so jolly sore all night;" or another would say, "I brought these flowers for you, Sister. I was down home yesterday." Or another shy boy would blush up to the roots of his hair and say "I thought perhaps you could use these fresh eggs for the children. My mother sent them. I was down with her for the week-end." And she would thank them with her swift glorious smile that made her such a favourite. You never saw a student laugh at the nurses in our ward, or in any way misconduct himself. They respected Sister too much, and were all too anxious to win her good opinion. When the doctor arrived Sister went always to his side, whilst he washed his hands, and many a wise hint she conveyed about the children who were very sick, and he was not above taking them. Meantime she had an eye for me, an eye for the dressing waggon, one for the children, or a new nurse.

"I will dress No. 4," Dr. S—— would say. And as I took off the child's shirt Sister would smile and say in a casual kind of way, "Harry won't be long before he gets away now, will he doctor?"

To which Dr. S—— would reply, "No I don't think so. I am glad to hear from you that he is now so brave."

And Harry feeling the centre of attraction, would press his lips together, determined to be brave after that remark. Dr. S—— was short-tempered if interrupted by crying or other noise. "Be quiet," he would thunder to a restless child, unheeding the tears that would rise at his words; but Sister would take the edge off his words with a smile and a gentle shake of her head, as much as to say "He does not mean it." So she contrived, and to this day I don't know how she did it, to keep surgeon, dressers and children all good humoured. For if they cried, as all children will do when hurt, they ceased whenever the dressing was over, and made less fuss than any other hospital children I have ever known.

### Nursing under the Municipality in Paris.

BY A LATE RESIDENT.

The Paris Municipal School of Nursing was created in 1877, and the first classes date from April 1878. Until this date the Paris hospitals had been nursed by nuns, that is to say, the sisters superintended and an untrained lay class worked under them in the wards. The nuns did not receive a hospital training, but commenced as superintendents. Having regard to what has taken place in France of late years, the introduction of lay nurses in the hospitals was bound to come, and since 1878 the nursing of 16 hospitals and asylums belonging to the city of Paris has been taken out of the hands of the nuns and given to the laity. It has naturally been a vexed question, those in favour of lay nurses contending that the nuns were ignorant and untrained, and would never submit to training and discipline, but must remain "irreconcilable enemies of civil society." The friends of the nuns had good grounds for protesting against the nursing of so many large institutions being taken from the religious communities, who for centuries had been devoted to nursing, "and given into the hands of a laity as yet untrained."

### What the Municipality Aims At.

Since 1878 the municipality has done a great deal for the training and education of its lay nurses, and it will be interesting for those who have not already studied the question to know at what they aim, and what standard they have set.

The École Professionnelle is municipal; it has a director, under the Department of the Assistance Publique; there are four centres for instruction, and examinations are held yearly, a diploma being given to successful candidates. But the diploma in France comes rather at the beginning than at the end of the hospital career. It can be taken at the end of the first year's work, but without it no promotion is possible; and, as soon as obtained, a nurse becomes a "suppléante," and is qualified to be an assistant superintendent, or superintendent of a ward, or set of wards. "Surveillante" corresponds to our sister; there are no female superintendents of nursing at the head of the French hospitals. Each has a director, who is responsible for the whole conduct of the hospital, and there is a "surveillante" over each "service," *i.e.*, kitchens, linen-room, wards, etc., so that each ward is practically worked as an independent unit.

### The Courses of Instruction.

The work required for the diploma is of a more or less preparatory nature. Two courses of instruction, one theoretical, the other practical, are given at each of the four centres, the classes being held from October to the following August. These classes are open to outside pupils as well as to the infirmières. The diploma requires a pass in seven subjects, *i.e.*, hospital administration, elementary anatomy, elementary physiology, hygiene, dressings and minor surgery, slight knowledge of chemistry, and the care of the lying-in woman and new-born infant. Pupils are also examined in practical subjects, which are taught by demonstrations, sometimes in the wards, sometimes in the lecture rooms. The practical work consists principally in bedmaking, temperature taking, a knowledge of instruments, bandaging and bandage making, hospital bookkeeping, and inventory taking. The classes are held from 8 to 9 P.M. twice a week, and the practical course is in the afternoons. Pupils from all hospitals must attend at one of the four centres. No term of residence, nor evidence of practical work in a hospital is necessary for the taking of a nurse's diploma. The outside, or "free pupil," who has never spent a day in hospital is equally eligible to stand for the diploma as the infirmière who has spent two or three years in the wards—nay, she has a much better chance of becoming a qualified nurse than the poor infirmière, who has been at work from 6 A.M. to 6 P.M., and is already tired and weary when she commences her studies at 8 P.M.

### Untrained Nurses with a Diploma.

Thus, while the École Professionnelle is answering its original purpose of educating the *personnel* of the hospitals, and raising the standard of their nursing, it is also turning out yearly a number of untrained persons, who have the

### NURSING UNDER THE MUNICIPALITY IN PARIS—*Continued.*

right to call themselves "diplomé" and to work as private nurses in the town. Even in the hospitals the nurses do not have a very varied experience, for as a rule a nurse remains in one "service" (ward or set of wards) until she rises to a higher grade. A superintendent told me that one of her nurses had been twelve years in her wards. Until the present time there has been only one class of infirmière in the French hospitals: that is to say, there have been no wardmaids, but all the rough work has been done by the nurses. There are "garçons de service" attached to most of the wards, for the heaviest work, such as lifting and removing patients, polishing the floors, cleaning the stairs and passages, but in all the hospitals which I visited the scrubbing of the floors and all the ward cleaning was done by the female nurses. With bad accommodation, rough living, and long hours, it is not surprising that so far it has been impossible to secure an educated class as nurses. Those in France who are interested in the progress of nursing quite recognise this, and better quarters are already being provided in some hospitals, and there is a strong movement to introduce a class of wardmaids; I was told in one hospital that they had already begun work there, leaving the infirmières free for the nursing. Under present conditions, as I have stated, the infirmière rises by taking her diploma, but there are many infirmières who are too uneducated to study for it, and recognising this the municipality has opened primary night schools in some of the hospitals where those in need of it can receive an elementary education.

### Male Nurses.

I have spoken of female nurses only, to avoid confusion, but a large number of the nurses in the Paris hospitals are male, most of the male wards being nursed by men, and they have the same chances of education as the women. Married as well as single nurses are employed, the married women being allowed to sleep out, and receiving some extra allowance for lodgings. The diploma, besides qualifying a hospital nurse for a higher post, entitles her to 3 fr. a month extra pay, until she gets promotion. Uniform, consisting of loose holland blouses, caps, and aprons are provided. Cuffs and collars are not worn, but a certain number of nurses wear over-sleeves, and all the male staff wear holland overalls.

### Attending a Lecture.

By the kindness of one of the lecturers, a lady doctor, I attended her evening lecture to the nurses at the Salpetrière. It was an interesting occasion as some half-dozen nuns were among the audience, the first time, I was informed, that any religious sisters had attended the municipal school course. The class numbered about 120 infirmières and outside pupils, all of whom listened attentively to a very clear lecture on the varieties of, and means of arresting, hæmorrhage, and dry and wet cupping. One could not but admire the ambition and eagerness for instruction of these infirmières—attendance at the lectures is voluntary—they had already done a long day's work, many had to come a considerable distance, and yet they were willing to undertake a course of study in the hope of gaining their diploma, for which in some cases they have to try several successive years. Most of the nurses I saw were of the class of girl to be met with in the little shops, or as domestics in small situations. The surveillantes struck me as capable women, intelligent and well educated in their work; they had evidently been deservedly promoted from the ranks of the infirmières.

### Retaining the Nuns.

In two of the Paris hospitals the nuns are still to be found, *i.e.*, the Hôtel-Dieu and the Boucicaut, and they also own most of the private nursing homes in the city.

Dr. Bourneville, director of the Municipal School of Nursing, has published a handbook of nursing ("Manuel Pratique de la Garde-Malade") in five volumes, which covers the course of the lectures for the diploma. For anyone interested in the question, volume 2 gives a full account of the schools and of the laïcisation of the hospitals.

### Beyond the Seas: Nursing in a Cawnpore Hospital.

BY AN OLD QUEEN'S NURSE.

The Zenana Hospital at Cawnpore was built and is supported by the Women's Mission Association in connection with the Society for the Propagation of the Gospel in Foreign Parts. It is called by the natives the "Zenana Hospital," as the poor suffering Indian women can come here without their one great fear of meeting a man. It was opened as a dispensary only in October, 1899, and for the first six months or so no in-patients were admitted. The staff consisted of one lady doctor, another being associated with her until the language had become familiar; one lady evangelist who acted as dispenser, both doctor and dispenser being assisted by a school-girl each from the mission orphanages near. Soon, however, they were joined by a nurse from England, who had previously enjoyed a long Indian nursing experience, so that the one available ward was quickly opened for in-patients. The other two wards were already taken up as dispensary and waiting-room respectively, while an inner room intended for nurses was used as a consulting-room. The picture shows the hospital in its unfinished state; there is now a long verandah or covered way running from it to the new dispensary, with the operation-room in the centre to the left.

### A Chapter of Disasters.

Before the nursing of in-patients could be fairly established, however, a serious outbreak of plague began, followed by the riots, which frightened all the patients away. When that panic had ceased, and peace was restored again, the lady doctor fell a victim to enteric fever, and the evangelist dispenser to a serious lung trouble. So the nurse was left to "run the hospital alone," which she did admirably, relieving the needs of all as far as lay within her power, until the lady doctor's return from the "hills," where she had spent the time of convalescence. Miss Wynne Edwards, L.R.C.P., and myself—I am an old Queen's District Nurse from Worcestershire and Huddersfield—came out in October, 1900, to repair the breach and replenish the staff. We found the lady doctor alone. The nurse had come to the end of her powers and was just starting for the hills. The evangelist had never recovered sufficiently to return, and to the sorrow of all concerned has never been able to do so for more than a few weeks, and then only to show a further development of the disease, and to be obliged to go back to her home in the hills, "Almora," the Bournemouth of India. We found plenty to do on our arrival beside learning the language. Certainly there were only two in-patients, but the out-patients were increasing in number, and for nurses I found two girls whose ages were only 16 and 14 respectively, one of them having only been in the hospital a month. Fortunately for us, when we reached Bombay we were entrusted by the managing directors with a girl of 16 who spoke English. So this girl accompanied us and acted as our interpreter. In a surprisingly short time she learnt dispensing; then, when her first year was up, she came into the wards to learn nursing. Now I call her my right hand, as she is the only one who can rise to an emergency, and "fly" if necessary to get things quickly. Her one shortcoming is that she cannot make her juniors do their share; she would sooner overwork herself than fall out with her comrades.

### Early Struggles.

I can never forget those early days, the strange weird look of an all but empty hospital, and the two child nurses, with their contempt for our English customs. "We have never carried away or washed bedpans, and we never will!" Such were the greetings I received from them on entering the ward in the morning to find a used chamber or bedpan under every bed and dirty spit-bowls everywhere. So I set to work doing the most menial things myself, to show them that work was honourable. I had to rush down in the morning to light the fire and make the beds. Bedmaking is one of the hardest things to teach, although so simple, in this country. The idea of the natives is that if sheets need shaking out or changing, cover them up and make all look tidy outside. I still remember the look of consternation and surprise on the faces of those girls when I stripped every bed, to find those in use dirty, and those not in use laden with skipping ropes, native spices and fruit. "How did the Mem Sahib know?" they asked, and they evidently thought me very clever that I had found out how they, with the help of another girl who worked in the dispensary, were just using the ward beds as cupboards for their playthings and food. Scrubbing tables and lockers was the next difficulty. The girls would persist in taking a jug of cold water and pouring a little on a table to be cleaned. Next they scrubbed it with a nail brush, then added a little more water, and so on until a few yards of floor were swamped with water and the table was finished! The same process applied to everything. I scrubbed and cleaned and made the girls do the same, until later I saw them look with pity upon a new comer who could not do these duties. Now I have about 12 girls who at least know something, and six of whom can be trusted to do night duty in turns, with a younger girl to help, although I always sleep near the patients on the verandah by the wards, so that the nurses can run to me in their least difficulty.

### Constant Exodus of Patients.

Our number of in-patients has steadily increased from the two or three we had at first to 25 or 30. But work cannot be estimated by these figures. Thirty here would be something like 100 at home, for the patients scarcely ever stay until they are convalescent. If a Mussulman woman comes in for a confinement it is because the "Dais" (native untrained midwives) "cannot manage"; they have done their best—or rather their worst—which often means that the case is hopeless, but if we are successful and the mother lives, then her husband and friends begin to worry to take her out the same day. Only the very strongest argument will induce them to allow her to stay until the third day. She will come back afterwards if things are not right, but she must return home to her people on the third day to go through some religious or other ceremony of their own. The task of admitting so many makes the work very hard. Often six or seven will come in in a day; by far the greater number of them reeking with tobacco, opium, and filth. We always

Exterior of the Zenana Hospital, Cawnpore.

### BEYOND THE SEAS: NURSING IN A CAWNPORE HOSPITAL—*Continued.*

with good reason, expect their heads to be alive with pediculi. "We do not bathe when we are ill!" said one high caste Hindoo to me, "we bathe when we are better." Quite half of those we admit remind me of the words of Naaman of old. "I thought the prophet would strike his hand over the place and heal it," but as to "wash and be clean," give up tobacco, opium, etc.; lie quiet in bed for a while: it is unheard of! "Sir Ghabrata" they often say to me. (My head is confused and worried.) "Ghar jana do" (let me go home). So many of them go, although they nearly always come back as out-patients; others by dint of much tact we are often able to keep, and sometimes we are indebted to old patients who know us, or to the chief woman of the tribe who stands and talks, reminding them that the Mem Sahib has done this and that with her own hand, therefore the patient should stop, and frowning she leaves the ward and the woman stays and recovers.

The Staff of the Zenana Hospital, Cawnpore.

### Our Girls.

Our new dispensary was completed and opened about a year ago. Miss Marval, L.R.C.P., came out at that time to fill the gap made by the first lady doctor's marriage with one of the S.P.G. missionaries here; Miss Wynne Edwards succeeding her as medical superintendent. Now one, and often both the doctors are busy in the dispensary (out-patient department) all the morning, six of the girls being told off to help, and very good they are at this work. The hospital being strictly on the Zenana system we cannot have a man or a boy on the place, and our girl nurses have everything to do. We are allowed, however, to have an "old man" to mind the gate. I wish hospital friends at home could see our girls at their work. Their ages vary from 15 to 18 or so; they are from the mission schools, and the greater number of them were made orphans by the famine or were deserted. They are all Christians now but some of them are only babes as far as Christianity goes and need the most careful looking after. Two or three, however, are exceptions, and to them we give the most responsible work. First we go into the consulting-room. Here a young girl is handing things to the doctor, as though she had never done any other work than this before, letting in and dismissing patients in turn, frequently explaining in better Hindustani the complaints of far away villagers whose dialects are quite beyond us, and who having "heard our name," to use their own expression, have come many miles to be treated. In the next room are two older girls doing dressings, preparing for, assisting at, and clearing up after minor operations with a skill and deftness which has often cheered me when discouragement has assailed me. . . . In the dispensary is our most trusted girl. She is dispensing medicine to from 30 to nearly 100 daily, except Sundays, two younger girls helping. She is only eighteen but has never been known to give incorrect medicine or doses. In the wards at this time the rough work is over for all the girls are being trained as nurses, dispensing coming afterwards as an extra to those sufficiently educated to learn. The day-workers begin in the wards at 5.30 A.M., and go to their appointed places in the out-patient department at about 8 in the winter and an hour earlier in the summer, leaving the latest probationers to look after the patients under my immediate supervision. So here about 10 A.M. the meal is being served by the Brahmin cook, and the probationers are helping the Christian patients, feeding the babies and the children of which we have a good percentage. The Brahmin is obliged to take the Hindoos' and Mahommedans' food to them as they would rather starve than take anything except milk, water, medicine, etc., from us.

THE CASES.

As to the kind of cases we have, they include nearly everything except leprosy and plague, and these two are sometimes found among the out-patients. But usually the patients are suffering from fevers, pneumonia, phthisis without count, diseased bones, rickets, bad eyes, cataracts, blindness, or syphilis. Starved, ill-fed babies, child-mothers scarcely able to stand, come for massage and rest; confinements and kindred troubles frequently show signs of the most shocking neglect. Here are a few of the last:—A little Hindoo girl I found on the verandah, a woman with her thrusting a parcel into my hand. To my horror the little girl's hand was wrapped up in it; it had been severed that morning by a train. We speedily took her in and treated her, and thankful we were to see her make a good recovery. The mother's one lamentation was, "Now I shall never get her married!" We recommend a mission school in such cases, and there, I believe, one or more of the children are attending. A beautiful high-caste woman came to us, two months after childbirth, with gangrene all down her left arm, the result of ignorant native treatment at home. Her pain was almost beyond endurance. We were very thankful to watch her recovery, although she lost part of her hand; for weeks we despaired of her life. Another case was that of a poor woman whom I found waiting her turn on the verandah, groaning in pain with a large tumour hanging from the labia; it had been coming for years, but had suddenly reached an enormous size. This the doctors were successful in removing. The woman stayed a week or so and went out thanking and blessing us. We have had many more, but these justify the existence of a hospital where women can be treated by some one else than a medical man who is only allowed to take a woman's pulse behind a curtain!

OUR NEEDS.

I feel sure that many at home may be glad to help us. Some have done so already. Our Society has dealt kindly with us, but we want much more. Six private wards were completed a year and a half ago, but for nearly a year now they have had to be closed and used for the nurses. Plans are prepared now for a suitable Home to accommodate 16 girls with two or three rooms for European workers to live with them, namely an English nurse, who assists me, and a working matron who helps with the stores and clothes, etc. There is also to be a room for the many who would like to come here as invalids or in the period of convalescence. We have only a room each in our own quarters. Then, too, we need badly a room for maternity cases and another for acute or dying cases. If a Hindoo or Mahommedan knew a patient died here she would instantly leave the hospital. Finally, any who are interested in the Cawnpore Hospital Building Fund should write to the Secretary, Women's Mission Association, care of the Society for the Propagation of the Gospel, 19 Delahay Street, Westminster, or to the Rev. F. Westcott, S.P.G., Mission House, Cawnpore, India.

### Charing Cross Hospital.

### NEW REGULATIONS FOR THE NURSING STAFF.

The nurses' new home at Charing Cross Hospital, described in the *Nursing Section* early in the year, is now complete. The building of the matron's office, and the permanent entrance in Chandos Street, have been finished, and the matron and her assistant, the home sister, are in occupation of commodious and convenient premises.

Concurrently with the reopening, on October 1st, of the hospital (closed since the middle of July on account of the structural alterations), certain new regulations concerning the nursing staff will come into force. These relate mainly to training, hours off duty, uniform, and scale of payment, and are the outcome of suggestions made to the Nursing Committee by the matron, Miss Mildred Heather-Bigg. They have received the approval of the Council, and are designed to bring the nursing department of this important institution somewhat more closely into line with those of the other great metropolitan hospitals.

To begin with, the length of the training has been extended to four years, with a view to securing an experienced staff from which staff nurses, night nurses, and sisters may be drawn as required to fill vacancies.

Women who wish to be received into the hospital for training as nurses must make application to the matron, a personal interview being indispensable. The matron sees candidates on Wednesdays and Fridays at 12. Candidates will not be accepted until they have been seen by a member of the medical staff, and they must be between the ages of 23 and 30, of average height, and single, or widows without children dependent on them. They will be accepted as candidates for one month's trial. If at the expiration of that time the matron considers them suitable they will remain for a further period of two months' trial. If accepted at the end of three months they will be expected to sign an agreement for four years. Probationers will be required to attend classes and lectures during their training as shall be directed. These, for first-year probationers, will be conducted by the matron and home and ward sisters, the latter giving practical instruction in the wards. To the ordinary work of bed-making, padding splints, bandaging, etc., it is hoped to add also massage and sick-room cookery. At the end of the first year's training (dating from admission) the probationers will be required to pass an examination in practical subjects. Until this is passed, probationers will not proceed to the training arranged for the second year, and if a probationer fails to pass, her engagement with the hospital will terminate, unless she is considered by the matron to be suitable in other respects, in which case she will be allowed to present herself again after six months. During the second and third years, probationers who have passed this examination will receive regular teaching from members of the medical staff in anatomy and physiology (second year) and surgical and medical diseases (third year). At the end of the third year they will be required to pass a second examination, before receiving a certificate of proficiency. No certificate will be given for a shorter period. Procedure to the fourth year of training is dependent upon passing this examination, and the same condition as to re-examination as in the case of that at the end of the first year holds good.

The rank of the nurses will be as follows:—First year, probationary nurses. Second and third year, staff probationers, the distinguishing marks being a hospital badge and uniform. Fourth year, probationary staff nurses, to be distinguished by a coloured waistband.

Staff nurses will be chosen from the ranks of the probationary staff nurses, and ward sisters from the staff nurses with not less than one year's experience of the

### CHARING CROSS HOSPITAL—*Continued.*

management of a ward by day or night, except under special circumstances approved by the committee.

Night nurses will, as far as possible, be drawn from third and fourth year nurses, their selection being determined by the nature of their ward reports.

With regard to hours off duty, there will be daily leave of absence for probationers for either two or three hours, one whole day a month, and three weeks' holiday during the year, two of which will be given at the end of the first six months, and the remaining week at the end of the year. After the first year the three weeks will be consecutive. Staff nurses will have four weeks' holiday, and sisters one calendar month, the times off duty of the former being two and a half hours daily, two evenings a week, and one day a month; and those of the latter two hours daily, from two o'clock to 11.30 every fortnight, and one whole day a month. Probationers not wishing to remain in the hospital may leave during the first month by giving the matron a week's notice.

The wearing of outdoor uniform is optional; probationers and nurses wishing to wear it must provide that chosen by the hospital, *i.e.*, a navy blue circular cloak, linen collar worn outside, and a navy blue straw bonnet with ribbon bows of the same colour. White strings are not to be worn. With regard to indoor uniform, aprons and caps only are given during the first year, probationers providing their own dresses according to the hospital pattern. In subsequent years dresses are provided. These are the property of the hospital, and are to be given up when a nurse leaves. Low shoes with rubber heels are always to be worn on duty; there is to be no wearing of jewellery, chatelaines, or buckles, and the hair is to be worn in a quiet style.

Some alterations have been made in the scale of payment, and the £3 "caution money," which has up to the present time been the custom, will be discontinued. First-year probationers will receive no salary; the payment for the second year will be £15; and that for the third year, £20; while for the fourth year, on passing the qualifying examination for probationary staff nurse, £26 will be given. Sisters' salaries will begin at £30, rising by annual increments of £5 to £45.

### The Prevention of Bed Sores.

### EXAMINATION QUESTIONS FOR NURSES.

Once more we come to the beginning of our examination year, and the improvement in the papers during the last twelvemonth and the ever increasing number of competitors give reason to hope that nurses find the work interesting and helpful.

Since this date last year a very great increase in the number of papers sent in by our colleagues across the seas has occurred. At first the response to our new departure in having a special paper for nurses in our Colonies and abroad generally met with but a lukewarm response, partly due, I think, to the matter not being quite clearly understood. Now, however, things are different, numbers increased in April last, and at the present moment a very respectable and encouraging pile of papers has accumulated for inspection and for judgment next month.

I should like to warn nurses in the United Kingdom to read the criticisms and remarks each month a little more carefully, and then they would not make the rather careless mistake of entering themselves as candidates among the Colonial nurses, and answering papers that are only to be judged six months hence. For nurses in our Colonies and elsewhere abroad, it is necessary to give a wide margin of time for the post, but a little thought would prove that as 24 hours is enough for a return post anywhere in England, Scotland, and Ireland, there would be no object in our allowing six months for consideration of the subject.

Last year I asked you all to remember that it is of the first importance that nurses should not confuse their sphere with that of the medical man. I will not repeat all I said then, but again impress upon you the fact that the best and most useful nurse is the one who obeys most implicitly the doctor's orders.

Never forget in giving reports of your cases to him, that you state facts and leave him to judge of them; do not be eager to give your own views. Your business is to state what has taken place in the absence of the medical attendant, but leave him to form his own opinion. An intelligent and truthful report is everything to a doctor and he knows how to value at her true worth a nurse who can give such a report and not be tempted to embroider thereon to enhance her own importance.

Once more I beg you to observe the rules. You would be surprised to know how many papers reach me without name or address, or with these important particulars written on a separate piece of paper, a proceeding strictly forbidden.

Fifteen days is allowed in which to answer the question, but answers frequently come to hand a month and five weeks behind time.

#### Question for October.

State what precautions you would take for the prevention of bed sores? N.B.—Do not enter on the subject of curative treatment, but confine yourselves strictly to prevention.

#### Reiteration of Subject.

The enthralling subject of the prevention of bed sores has been considered before, but it is unavoidably repeated now, to coincide with the question given to the nurses in the Colonies, etc., as it is interesting to compare the views of all.

The Examiner.

#### Rules.

The competition is open to all. Answers must not exceed 500 words, and must be written on one side of the paper only. The pseudonym, as well as the proper name and address, must be written on the *same* paper, and *not on a separate sheet.* Papers may be sent in for fifteen days only from the day of the publication of the question. All illustrations strictly prohibited. Failure to comply with these rules will disqualify the candidate for competition. Prizes will be awarded for the two best answers. Papers to be sent to "The Editor," with "Examination" written on the left-hand corner of the envelope.

N.B.—The decision of the examiner is final, and no correspondence on the subject can be entertained.

In addition to two prizes honourable mention cards will be awarded to those who have sent in exceptionally good papers.

### To Nurses.

**We invite contributions from any of our readers, and shall be glad to pay for "Notes on News from the Nursing World," or for articles describing nursing experiences, or dealing with any nursing question from an original point of view. The minimum payment for contributions is 5s., but we welcome interesting contributions of a column, or a page, in length. It may be added that notices of appointments, entertainments, presentations, and deaths are not paid for, but that we are always glad to receive them. All rejected manuscripts are returned in due course, and all payments for manuscripts used are made as early as possible after the beginning of each quarter.**

### Echoes from the Outside World.

### Movements of Royalty.

The King has been quietly enjoying his holiday in the Highlands. Attended by several members of the Court he has been frequently out in his motor car, and on Monday he called on the Princess of Wales, afterwards going to Mar Lodge to have lunch with the Duke and Duchess of Fife. The Royal party subsequently proceeded to the Linn of Quoich, where tea was served. Amongst other visitors to the Castle is Admiral Sir John Fisher, Commander-in-Chief at Portsmouth. He is expected to remain for a week, during which time the King wishes to discuss with him full details of the arrangements for the reception of the King and Queen of Italy on November 16th. His Majesty's yacht, the *Victoria and Albert*, will cross over to Cherbourg for the visitors, and they will be escorted by four ships of the cruiser squadron, whilst in mid-channel they will be met by eight vessels forming the Portsmouth instructional flotilla of torpedo-boat destroyers. The Channel Squadron will assemble at Spithead and join in the reception of their Majesties who will stay in London three or four days.

Perhaps partly owing to the fact that Queen Alexandra will of necessity be in England to receive her visitors, the King of Denmark, contrary to expectations, has refused to celebrate in any marked manner the fortieth anniversary of his accession to the throne which falls on November 15th. His Majesty desires to pass the day quietly in the midst of his own family. Although King Christian IX. is now in his eighty-sixth year, he says that it will be time enough to have public celebrations when he has ruled his land for fifty years.

### Queen Victoria's Letters.

It was officially announced on Monday that the King had commanded the publication of selections from the correspondence of Queen Victoria between the years 1837, when she succeeded to the throne, and 1861. The work will be edited by Mr. Arthur Christopher Benson, son of the late Archbishop of Canterbury, who is a master at Eton, and Viscount Esher, the well-known Deputy-Governor of Windsor Castle. This announcement explains the presence at Balmoral of Lord Esher, who at the beginning of last week was said to have visited the King in connection with political business.

### The Crisis in the Balkans.

The Prime Minister has replied at length to a letter from the Archbishop of Canterbury expressing the growing necessity among Churchmen lest any steps should be omitted which might diminish the sufferings of the Macedonian population. After expressing his sympathy with "the feelings of horror and of indignation which the present position of affairs in South-eastern Europe must excite in the heart of every humane man," Mr. Balfour points out the peculiar difficulties and complications of the situation. He states that in the opinion of the Government the best chance of dealing with them successfully lies in the continued co-operation of Austria and Russia. From this it follows that our best hope at present of ameliorating the condition of Macedonia, as well as of avoiding international complications, is to support the two Powers. This, Mr. Balfour says, does not preclude the offering of suggestions, and he adds that the Government have offered them, and will continue to do so when fitting opportunity presents itself. In a communication addressed on Saturday to the Bulgarian Government by the British representative at Sofia, it was intimated that the British Government was pressing the Porte to apply the Austro-Russian reforms in a more efficacious manner.

### Death of the Duke of Richmond.

By the death of the Duke of Richmond and Gordon, K.G., at Gordon Castle, on Sunday, the country loses one of its best known peers. The Duke was in his 86th year and succeeded his father in the family honour and estates in 1860. A year previously he was President of the Poor Law Board, an office he filled again, after his accession to the title, in 1867–68. He was subsequently President of the Board of Trade, Lord President of the Council, and Secretary for Scotland, retiring from office finally in 1886. In 1887 he lost his wife, to whom he had been married 44 years, and he was never quite the same man after her death. His eldest son, the Earl of March, now in his 58th year, becomes Duke of Richmond and the owner of nearly 20,000 acres in Sussex, including "Glorious Goodwood," as well as the great property in Scotland consisting of nearly 270,000 acres, on which there are said to be some 900 tenants. The late Duke will be buried at Goodwood on Saturday.

### The New Lord Mayor of London.

At a meeting of the Court of Aldermen in the Guildhall on Tuesday, Alderman Sir James Thomson Ritchie, head of the firm of Messrs. W. Ritchie and Sons, jute merchants, Lime Street, was elected Lord Mayor of London for the ensuing year. Seven names were submitted to the Liverymen, but only those of Sir James Ritchie and Mr. Alderman John Pound to those of the Court of Aldermen. The Lord Mayor Elect was born in 1835, is a native of Scotland, and elder brother of Mr. C. T. Ritchie, who has just resigned the Chancellorship of the Exchequer, to whom he bears a strong resemblance. In 1892 he made an unsuccessful effort to enter Parliament for the City of London as an Independent Conservative. A few years ago Sir James Ritchie had to contradict the announcement of his own death.

### Mr. Barrie's New Play.

The clever author of "Little Mary" at Wyndham's Theatre describes it as an "uncomfortable" play. But the only discomfort which the audience seem likely to experience is that which occasionally arises from uncontrollable laughter. The prologue shows a little girl of twelve, Moira Loney, living with her Irish grandfather, Terence Reilly. Whilst the old chemist has been busy writing his pamphlet on how to cure the English nation of illness—it has grown to three massive tomes—his grandchild has indulged her motherly instincts by taking four motherless infants to look after in her spare time. Lord Carlton coming to the shop to purchase headache powders makes the acquaintance of this strange little mother, and the conversation which ensues, interrupted by sundry infantile heads which pop up from impromptu cradles at odd times, is extremely entertaining. Six years elapse before the next act. The grandfather is dead, and Moira Loney, now known as "The Stormy Petrel," is busy curing many folks of many diseases by the aid of the medium "Little Mary," of whose powers she has learnt in her grandfather's book. By the end of the second act the secret of the cure is disclosed, and Lord Carlton has also succeeded in persuading "The Stormy Petrel" to become Lady Carlton. The play is admirably acted, the burden of the representation falling principally upon Miss Nina Boucicault, who fulfils her obligations with unfailing skill. Mr. John Hare as Lord Carlton shows his usual polished incisiveness. Mr. Gerald Du Maurier and Mr. Eric Lewis are satisfactory as Lord Rolfe and Sir Jennings Pyke. Mr. Harry Vileart gives a pathetic rendering of the old grandfather, and Miss Margaret Fraser's Eleanor Gray deserves mention.

### A Book and its Story.

NEW NOVEL BY MARY FINDLATER.*

The author of "The Rose of Joy" has chosen the life story of her heroine, Susan, as a practical illustration of Emerson's teaching, contained in the following lines, which are found on the title page:—

"In the Actual—this painful kingdom of Time and Chance—are care, canker, and sorrow, With Thought, with the Ideal, is immortal hilarity—'The Rose of Joy'—round it all the muses sing."

The story is extremely interesting in a quiet way. It has power, pathos, some humour, and is healthy in tone. The character of Susan holds the reader's attention throughout, and the more brilliant characters fail to attract beside it. It is one with a rare capacity for self-sacrifice, full of sympathy and unfailing patience. To such an one life is full of opportunities for the use of these virtues. She is not long in finding scope for their exercise. Her father dies, leaving her with several brothers and sisters younger than herself. Her mother is described as being, "when she married, possessed of a fresh complexion, good teeth, and abundance of hair, also a pretty power of blushing and sitting silent. By thirty-six her complexion had lost its bloom, and her hair was thinning fast. At fifty, hair, complexion, and teeth were alike gone; her husband was gone too. She could no longer blush, but she could still keep silent because she had nothing to say. What few accomplishments she had laid on in her youth were long since worn away—gone as completely as the plating off an old spoon—but seven children all living, formed her solid contribution to society." To Susan fell the rôle of general assistant and condoler to the household. Beloved by the boys and girls, not understood by her mother, with whom she had little in common, her days were passed in the monotonous routine of family life. But she had compensations. When the evening came she retired to her room and then she "found" herself. "In the cold damp room that she shared with her sister she had thrown a jacket over her shoulders to keep off a chill and sat down at a table. Her eyes gleamed like a person who anticipates some great pleasure. The day was over, her own interest had begun. She took out from the table drawer a thick, shabby drawing-book that was filled almost from cover to cover with drawings, some in water-colour, some only pencil sketches. The larger pictures showed more attempt at design than the smaller. They were remarkable to anyone who could judge of their work, from the total absence of hesitation. Out of drawing and grotesque as many of the figures were, they moved, they stood firm on their feet, they held out hands to each other with just that ingenuous power, that humble feeling after truth, recognisable everywhere as being lit by 'that divine spark which no industry can ever kindle, which no neglect can ever quite destroy." Susan also read a great deal. Modern books did not appeal to her and "classics do not foster self-consciousness." So she never thought of herself, except unfavourably, and had no idea that she was in any way unlike other people. "There was nothing of the self-pity or self-absorption of the young genius about her. She had a very simple heart."

Susan had no beauty to commend her. She was only a heroic little girl whose outer and inner life were disconnected. The day's duties were performed patiently, "whilst all the while her heart was busy with its own occupation—one which no one she lived with could share." Whilst Susan was dividing her time between working and dreaming, she was pleasantly startled by the announcement of an invitation to stay with some old friends of her mother's. Her sister was to accompany her. Having never left home, the thoughts of the possibilities of enjoyment held out by the visit were delightful. The invitation was accepted, and upon their arrival at Linfield, Susan found much to satisfy and soothe the artistic perceptions which, at home, were in a constant state of irritation from their uncongenial environment. Here, "for the first time in her life, she found herself in a house that satisfied her sense of beauty. . . . . 'There is nothing ugly,' she thought, and whenever her eye lighted on anything she felt a little new soft shock of delight. Before she had been five minutes in the house her consciousness was sensibly enriched . . . to Susan it seemed as if after discord, life had suddenly glided into harmony. Her whole personality in a day or two began to unfold as a blossom opens in a blander air." She, lost in the charm of her new surroundings, was unconscious of the impression she was making upon her hostess, and her hostess' brother, Colonel Hamilton. "'She is not in the very least like what Maria used to be,' Mrs. Clephane remarked when the girls left the room. 'Maria was so pretty!'"

"'She is not pretty in that kind of way,' he answered; 'but I think she has a face "that serves the ends of beauty," as someone puts it.' This was a little beyond Mrs. Clephane's comprehension, who thought Susan a very plain-looking little person, and saw nothing absolutely about her to admire."

Susan, in spite of this, managed to win all hearts, even that of Juliet, the lovely niece of Colonel Hamilton, who lived with her mother at Linfield. Juliet was charming, but not "inseeing" like Susan. She was troubled by Susan's indifference to dress, and after a shopping expedition she returned in despair to confide to her uncle that Susan was always running away from the matter in hand. "And what is the matter in hand?" "Why to get her decently dressed like other people, of course." "She's so sweet as she is, I think." "Yes, so she is; but she'd be much sweeter if she were just a little different." "I wonder if she would," he replied.

At an exhibition of pictures in Edinburgh Colonel Hamilton comes upon the two girls. "Juliet, who cared no more for pictures than she cared for astronomy, went merely because other people did." Not so Susan, who was lost in contemplation before one of the old Italian masters. To Juliet there was only incongruity, absurdity almost, in the pose of the figures. After a pause, Juliet turns again to ask where the charm lies, and continues: "Well, what do you see in it?" "The intention," said Susan seriously. "Is it not that, after all, which makes everything good or bad?" Colonel Hamilton, coming up, overhears Susan's remark. He went up quickly to them. "You understand, I see," he said, and Susan, a little shyly, answered, in the words he had used to her at another time, "These are the indestructible joys for ever."

Juliet turned away to speak to someone she knew. She had an uncomfortable feeling that here was a matter which she knew nothing about at all. At Linfield, Susan makes the acquaintance of Darnley Stair, "an ugly young man, with some distinction of manner. In spite of his carrot-red hair and his plain face, there was a kind of grace about the creature every now and then; there were both humour and feeling in his eyes." He is openly an ardent adorer of Juliet, who disdains his attentions frankly. Susan returned home, and shortly afterwards goes to live with her aunt and uncle Murchison.

Mr. Murchison is a flourishing brewer, and Mr. Stair is his pupil. Her life at home had become so wearisome that her aunt had insisted on keeping her with her. She sees a good deal of Mr. Stair, and ends by marrying him. After marriage her life lacks neither incident nor excitement. This part of the book is by far the most clever. How poor little Susan tries to take up the broken threads of a shattered life and weave them into some coherence, we must leave our readers to discover in the book themselves.

* "The Rose of Joy." By Mary Findlater. Methuen. 6s.

### Everybody's Opinion.

### NURSES AND PUBLIC WORSHIP.

"One in Sympathy" writes: A nurse in a small fever hospital in Fifeshire was asked the other day through the medium of a postcard to call at the church treasurer's place of business. On doing so she was informed by him that a member of the congregation who had a seat in the pew (used by three of the hospital staff for three years) objected to the smell of disinfectants employed by them. This is very keenly felt by the nurses, who during the three years have become attached to the church and its minister. If by inserting the above you can enlist the sympathy of your readers for those in a similar position you will do a great deal to brighten the lives of those who for the sake of doing their duty give up so much.

### A CANCELLED APPOINTMENT.

"Inquirer" writes: I should like to have the opinion of others on the following case: I was recently appointed matron to a small hospital and training home after an interview with the lady to whom application was to be made. As the committee were desirous of the post being filled as soon as possible the decision appointing me to the post was wired to me, so that I might resign my present post at once. A letter came the following day confirming my appointment. On receipt of the telegram I at once sent in my resignation that no time might be lost. Twenty-four hours after another telegram came cancelling the appointment, as circumstances rendered it necessary. This telegram also was followed by an explanatory letter, adding that this action in no way reflected on my qualifications. I at once cancelled my resignation, thereby having to ask leave to remain in my present post. The salary in the new appointment was better than that of the present one. I wonder if I can ask any compensation in such a case, as it seems to me a distinct breach of contract.

[If, in consequence of your engagement by the committee, you had been thrown out of an appointment you would have had cause for making a claim against them. But as you have only suffered prospective loss you could not sustain an action for breach of contract.—Ed. The Hospital.]

### BICYCLES FOR DISTRICT NURSES.

"Miss Katharine H. Greg" writes from Lode Hill, Handforth, Cheshire: Those of your readers who applied to me for a bicycle which I offered to give to a district nurse—through an August number of The Hospital—may be interested to hear the result of their appeal. I received no fewer than 60 applications, and it may be imagined what a difficult task I have had in deciding among so many. The thought of disappointing so many also caused keen regret, and I determined, if possible, to procure some more bicycles, and to this end I wrote a letter to a Manchester newspaper requesting any ladies who had bicycles to spare to communicate with me. This letter brought me various replies, one from an invalid lady who was so much pleased with the idea of being able to help nurses in their work that she asked me to buy two bicycles at her expense, and to forward them to some of my applicants. I also received other offers of bicycles which have already seen good service but are considered capable of still further work. These I have despatched after very careful consideration of the appeals, and with the sincerest desire to help the most urgent cases. To those whose demands I have unfortunately not been able to satisfy I would suggest that they, or their committee, should ask the families in their neighbourhood whether they have any spare or discarded bicycles, for I feel sure that many ladies must have sufficiently good cycles put aside which they would gladly give to a district nurse if only the request were made. I may mention that if I should happen to receive any further offers of bicycles I shall be only too pleased to forward them to the addresses of the nurses I already have, and I am glad to think that I have been able to provide seven of them already with machines. It seems little enough that the outside public can do to lighten the labours of the district nurse, but we assuredly appreciate their good services, and would wish to give them every help and encouragement possible.

### Appointments.

**[No charge is made for announcements under this head, and we are always glad to receive, and publish, appointments. The information to insure accuracy should be sent from the nurses themselves, and we cannot undertake to correct official announcements which may happen to be inaccurate. It is essential that in all cases the school of training should be given.]**

Bedford Isolation Hospital.—Miss Charlotte Kenderdine has been appointed nurse in charge. She was trained at St. George's Hospital, London, where she was afterwards nurse. She has since been superintendent nurse at Fulham Workhouse Infirmary, and Bedford Workhouse Infirmary.

Bromley Cottage Hospital.—Miss Gertrude M. Allen has been appointed senior staff nurse. She was trained at Guy's Hospital, and has since been nurse in a surgical home in London and sister at Bartholomew's Hospital, Rochester.

Cardiff Workhouse Hospital.—Miss Louie Jones has been appointed charge nurse. She was trained at the Fir Vale Infirmary, Sheffield.

Derby Union Infirmary.—Miss Margaret Fegan has been appointed staff nurse. She was trained at the Hackney Union Infirmary, where she afterwards became staff nurse, and has since been staff nurse at the Colchester Union Infirmary.

East Poorhouse Hospital, Dundee.—Miss Matilda Nicol Kidd has been appointed charge nurse. She was trained at Leeds Union Infirmary, and has since been attached to a private nursing home in Manchester.

Incorporation Infirmary, Shirley Warren, Southampton.—Miss Annie Dowbiggin has been appointed matron, and Miss Florence L. Cross sister. Miss Dowbiggin was trained at the Leeds General Infirmary, and afterwards held the post of charge nurse at the Park Fever Hospital, London; theatre sister at the Royal Hospital, Portsmouth; head sister at Moseley Hall Convalescent Hospital, Birmingham; and assistant matron at Shoreditch Infirmary, London. She has the L.O.S. certificate. Miss Cross was trained at Shoreditch Infirmary, and has since been staff nurse at the same institution.

Jaffray Branch Hospital, Erdington, Birmingham.—Miss Jessie Greening has been appointed sister. She was trained at the Children's Hospital, Birmingham, and the General Hospital, Northampton.

Oakwell Infectious Hospital, Yorkshire.—Miss Annie Newbold has been appointed matron. She was trained at the City Infectious Hospital, Nottingham, and at the District Hospital, Batley, Yorkshire. She has since been charge nurse at the Milton Infectious Hospital, Portsmouth, charge nurse at the Borough Hospital, Croydon, temporary nurse at the Basford Sanatorium, Nottingham, and matron at the Infectious Hospital, Foleshill, near Coventry,

Sheffield Union Infirmary.—Miss Elizabeth Emily Jackson has been appointed home sister. She was trained at the Royal Infirmary, Bradford, where she has since been sister of the male medical wards and housekeeper.

Sheffield Union Infirmary.—Miss Kate Sophie Johnson has been appointed sister. She was trained at Bradford Union Hospital, and has since been engaged as private nurse.

Thos. Walker Hospital, Fraserburgh, Aberdeenshire.—Miss Annie Hepburn has been appointed nurse-matron. She was trained at the Royal Infirmary, Glasgow, and has since been on the staff of the Royal Scottish Nursing Institution, Edinburgh.

West Ham and East London Hospital.—Miss Florence Smith has been appointed sister of the women's ward. She was trained at Gravesend Hospital, and has since been staff nurse and receiving-room nurse in the West Ham Hospital.

### Novelties for Nurses.

By Our Shopping Correspondent.

### TRISCUIT.

Triscuit is a biscuit formed of the whole grains of wheat, cleaned, filamented, formed and baked, at the Niagara Falls, by electricity. Being made of whole wheat it is of course highly nutritious. It may be used as a substitute for toast, or triscuit may be used as a basis for other dishes. Triscuit is convenient to carry where food supplies are doubtful. Triscuit is very dry, and it is necessary that some fluid should be available if it is to be used. It is sold by all grocers.

### PINE-WOOL GARMENTS.

The Knitted Corset and Clothing Company, of Nottingham, are manufacturing a variety of pine-tree products, amongst which are materials for underclothing, which are of very fine quality. The aim of the company is to supply garments beneficial to rheumatic persons. The soft and warm materials, fragrant with a most pleasant odour of pine, certainly commend themselves most highly for the purpose intended. The material seems an ideal one for winter wear, and those about to purchase winter garments would do well to send to Nottingham for a catalogue before deciding to go elsewhere. The dress materials in pine-wool are also excellent.

### PERI-LUSTRA.

Peri-Lustra is the name given to a series of most attractive yarns, made of cotton in such a manner as most closely to resemble silk. They are an excellent example of what can be effected with cotton through the modern process of "mercerisation," and peri-lustra, at a much lower cost than silk, bids fair to supersede for embroidery, knitting, or crochet where silk has hitherto been employed. Amongst the examples of peri-lustra before me, the most admirable appears to me to be that described as "stout embroidery for art needlework." Its brilliancy, softness, and firmness render it especially desirable for the purpose. The crochet yarn, knitting yarn, embroidery thread for flannel and other purposes, and the flossette flourishing thread are all to be praised. Peri-lustra is made in numerous and beautiful shades of colour, and can be obtained at all fancy-wool shops.

### HYGIENIC UNDERWEAR.

I have examined a number of samples of Dr. Rasurel's new hygienic underwear, which, from their claim to being antiseptic, absorbent, and unshrinkable, demand special attention. The antiseptic and absorbent properties are claimed to result from the introduction of peat, and therefore the use of such garments is specially recommended in rheumatic cases. Apart from any special recommendation the exquisite quality of Dr. Rasurel's textures places them in the foremost rank of woollen materials for underwear, and the price is very moderate, having regard to the quality of the clothing. The grades in relation to warmth commence with the most delightfully soft, light, and elastic netting—an ideal substance for wear in tropical climates. This grade is followed by others more close in texture but retaining the fine qualities of the first I mentioned. The warmest quality, a fine stockingette with a reverse side of the silkiest down, is so warm and soft that it should meet the requirements of the most chilly mortals. Every variety of garment for man, woman, and child can be secured in each quality and in numerous sizes, and all are well made and carefully finished. The agents are the Nurses' Outfitting Association, Stockport.

### UNDERWEAR OF SOFT COTTON MATERIALS.

There are so many things to be said in favour of elastic cotton fabrics for undergarments that it is not to be wondered at that they are increasing in popularity. Dr. Lahmann has taken advantage of all possibilities of the substance to produce the most delightful material for which it may confidently be claimed that it is soft, durable, and inexpensive. It is also much more economical than a woollen material, which when made into fitting garments usually has to be discarded long before it is worn out because it has shrunk in cleaning. This quality in connection with woollen garments serves to recommend cotton from a hygienic point of view. Many people, knowing the deterioration in wool garments through the cleansing process, wear them longer than they should do. Cotton does not lose its elasticity, and therefore there is no hesitation necessary in submitting it to the laundress. Dr. Lahmann's cotton wool material is made in numerous grades of thickness, for use in the warmest summer or the coldest winter weather. It is fashioned into every variety of men's, women's, and children's garments, and may be obtained at most drapers or from the agency, 15 Fore Street, E.C.

### MESSRS. EGERTON BURNETT.

The arrival of a well-filled box of patterns from Messrs. Egerton Burnett reminds one that summer has departed, and that we must turn our thoughts to autumn costumes, and take a general review of our wardrobes. With regard to dress materials, the only difficulty lies in selection from the extremely varied assortment before us. Bearing in mind, however, the fact that the sensible and useful coat and skirt is still fashionable and likely to retain its popularity, it will be wise to choose some good, serviceable material from among the tweeds, friezes, zibelines, hopsacks, and other fabrics for this purpose. These are mostly of quiet shades, some of the nicest having a black or dark grey ground with silky or hairy-coloured knots, which this year, I notice, run in stripes along the length of the stuff. I especially like the fancy flake coating, styled "The St. Albans," which has these stripes and knots in blue, green, or black on a dark ground. It is 44 inches in width, and costs 3s. 9d. per yard. The same idea is carried out in the "Clarendon," which is somewhat cheaper, and made in more varied colours. The "Downing" has, in addition, white spots woven into the fabric, and these are accompanied in one of the patterns by flakes of some shining thread; while the many varieties of costume cloth—"Dolgelly," "Onslow," "Edina," and others—are pervaded by knots and stripes. I believe a good hopsack wears as well as anything, and there are some charming patterns in black, navy, or electric blue, brown, green, red, and fawn. They are 50 inches wide, and 3s. 3d. per yard. Then for wearing under the skirt, the Park Lane skirtings are something new and tasteful, admitting a choice of colour to match the stripe in the dress.

Messrs. Egerton Burnett inform me that owing to the increasing favour in which their tailoring department is held by their clients they have recently considerably extended their premises, and that having an efficient staff they have every confidence in asking their customers to fill in the self-measurement forms and have their dresses made at Wellington. A register of measurements is kept in order to avoid the necessity of sending them time after time. The designs shown me are neat and pretty; and the "Egerton" cycling costume, from 31s. 6d. (or skirt only, 12s. 6d.), is especially nice.

A word as to uniforms. This firm is, as readers of The Hospital are well aware, the holder of more than twenty Royal and Imperial warrants of appointment, and their Royal serges, of pure wool and British manufacture, are of world-wide fame. The price of the navy blue serge—one of the most useful and necessary of dress fabrics for nurses—varies from 1s. 11½d. to 6s. 11d., and is of double width, while the waterproofed serges, for out-of-door wear in the form of dresses or cloaks, are from 3s. 3d. to 5s. 11d. Then I need hardly remind my readers that most if not all of the cottons and galateas required for hospital dresses are supplied by this firm, whose long experience in the manufacture of uniform materials is thoroughly well deserved. Nurses' cloaks, tailor-made, thoroughly waterproofed, and specially dyed to prevent their turning a bad colour, are made to measure, and patterns and prices for special shapes will be sent on application to Messrs. Egerton Burnett, Wellington, Somerset.

### Presentations.

St. Mary's Hospital, Manchester.—Miss Tibbits having resigned the matronship of St. Mary's Hospital, Manchester, which she held for eleven years, was presented by the board of management and honorary staff with a handsome gold presentation watch, expressing their regret at her resignation and appreciation of her services. The nursing staff, past and present, gave a long gold chain with cross attached. The present and two late house surgeons a gold and pearl curb bracelet. The servants gave silver teaspoons and butter-knife. Miss Tibbits will be much missed and regretted by all at St. Mary's Hospital.

### "The Hospital" Convalescent Fund.

The hon. sec. begs to acknowledge with thanks the receipt of £1 from a Bristol friend who wishes to remain anonymous.

### Where to Go.

Tuesday, October 6.—At 8.30 p.m., Lecture, by Rev. J. B. Booth, on "Ruskin," at the Central Institute, 26 St. George's Street, Hanover Square.

### TRAVEL NOTES AND QUERIES.

Pensions in Rome (E. C.).—Please remember that a pseudonym is necessary. I hope however this will catch your eye. First hotels. Hôtel Eden, 49 Via Ludovisi; this is near the Pincio, and is very sunny. Terms from 8 fr. per day, but if you remain a long time you can generally make a favourable arrangement, probably 6 fr. or 6 fr. 50c., only it is important to remember that the lower terms always mean less good bedrooms, because the food is the same to all, therefore it is well to mention that a sunny bedroom is indispensable. Then Hôtel du Sud, 23 Via Lombardia, from 7 fr.; Hôtel Poste, south of the Piazza di Spagna, from 8 fr. Now pensions. Avanzi, 75 Via Capo le Case, from 6 fr.; Union, 121 Piazza di Monte Citorio, from 6 fr.; Hurdle-Lomi, 36 Via del Tritone, from 8 fr.; Miss Hayden, 42 Piazza Poli, from 8 fr. I have had a very good account of the Avanzi, but it is some little time since, and, like other cheap pensions, may have changed hands, but I should be inclined to write and inquire. If I can do anything further for you let me know nearer the time. If it is your first visit you would enjoy it much more if you get up your subject thoroughly by reading Hare's "Walks in Rome," Mrs. Elliott's "Roman Gossip," Storey's "Roba di Roma," Hawthorne's "Transformation," Ouida's "Friendship," and "Ariadne."

### Notes and Queries.

### REGULATIONS.

**The Editor is always willing to answer in this column, without any fee, all reasonable questions, as soon as possible.**

**But the following rules must be carefully observed:—**

**1. Every communication must be accompanied by the name and address of the writer.**

**2. The question must always bear upon nursing, directly or indirectly.**

**If an answer is required by letter a fee of half-a-crown must be enclosed with the note containing the inquiry.**

*Translation.*

(1) I shall esteem it a great favour if the Editor of The Hospital will kindly reply to her questions, *i.e.*, by translating the following:—1. Pace tanti viri. 2. Quinimmo non pauci ex iis viris qui vitam degentes solitariam chartis solent impallescere eodem morbo tentantur.—*Nurse K.*

1. By the leave of so great a man. 2. Moreover, not a few of those men who are wont to grow pale leading a solitary life with books, are troubled by the same disease.

*Home.*

(2) Will you tell me of any institution which would train a girl of eight, who has lost her right arm, to some useful occupation? Her parents could afford to pay a small sum weekly.—*Nurse G.*

Write to the Invalid Children's Aid Association, 18 Buckingham Street, Strand, W.C.

Can you tell me where a semi-invalid could be received on reciprocal terms? She has a decided taste for nursing, and is always able to do some work, having nursed her mother through a long trying illness. She is 30 years of age.—*C. E. G.*

Possibly you may obtain a home for her by advertising.

Can you tell me of any school where a blind lady, also slightly deficient mentally, could be received for a small yearly payment?—*N. D. Sister K. E. R.*

You do not mention her age. See Charities for the Blind in "Burdett's Hospitals and Charities."

Can you tell me of any home near Cavendish Square where a gentleman partially paralysed, could be received permanently, for 21s. a week?—*D. I.*

A few private patients are received at the Hospital for Epilepsy and Paralysis, 4 Maida Vale, W. An advertisement would probably bring you some replies.

I am anxious to find a home for a poor girl of 17, paralysed from the waist downwards. Is there any charitable institution to which she could be admitted free of charge, or where only a small fee would be required?—*L. G. I.*

You might make application to the National Hospital for the Paralysed and Epileptic, Queen Square, Bloomsbury, W.C.

*Signed Articles.*

(3) I want to know if the names of your contributors are kept secret. I wish to write an article shortly, and I do not want my real name to transpire.—*Unsigned.*

Contributors' names are never divulged unless they wish it.

*Maternity Work.*

(4) Is there any nursing home where a maternity nurse, without general training, could be received?—*Nurse C.*

Possibly by advertisement she might find a place on the staff of a private home.

*Superfluous Hair.*

(5) Will you kindly tell me of a safe and inexpensive remedy for superfluous hair on the face?—*C. G.*

Kindly tell me the treatment for removing permanently superfluous hair from the face, and the nearest place where it can be obtained.—*Lancashire.*

Hair can only be removed permanently from the face by electrolysis, which is fairly expensive. The treatment is chiefly carried on by toilet specialists, but as it is a surgical operation, it is better to consult a medical man.

*New York.*

(6) Are there any hospitals, general or other, in New York, in which a certificated nurse could obtain a post as charge nurse? I should be much obliged if you could send me the addresses of such. *Omega.*

You will find a list of the more important hospitals of New York in Burdett's "Hospitals and Charities." Probably a personal interview would be necessary.

*Cairo.*

(7) Can you tell me if there are any private nursing institutions, or homes at Cairo, and give me any information concerning them?—*H.*

We do not give information concerning private institutions, but the nursing staffs of the Station Hospital, the Citadel, and the Kasr-el-Ainy Hospitals are under the directions of English nurses, who might be willing to advise you on the subject.

*Visiting Nurse.*

(8) Will you give me the address of any nursing institution which has inaugurated daily visiting to middle-class patients, and say what terms are charged per visit.—*M. D. F.*

Nurse Thomas, 10 Montagu Place. W., would give you particulars of the Marylebone Visiting Nursing Association. The fees are from 3s. 6d. per visit.

*Uniform.*

(9) I am a mental nurse of two and a half years' standing My patient wishes me to wear uniform. Would I be doing right if I complied with my employer's wish?—*An Inquirer.*

Certainly.

*Massage.*

(10) Kindly say if there is a place in Liverpool where I can take lessons in massage.—*B. T.*

There is no public school of massage in Liverpool. Write to the Secretary, the Incorporated Society of Trained Masseuses, 12 Buckingham Street, Strand, W.C., and ask her to recommend a private teacher.

*Sanitary Inspector.*

(11) I should be grateful for any information concerning lady sanitary inspectors. Is it a wise thing to undertake?—*Nurse Jessie.*

Write to the Secretary, The Sanitary Institute, Margaret Street, W., for full particulars. There is a fair and growing demand for thoroughly competent persons.

*Maternity.*

(12) I am anxious to train as a monthly nurse, but I cannot afford a high fee.—*A. S. E.*

A list of lying-in hospitals with their terms in full will be found in "The Nursing Profession: How and Where to Train."

*Plague Nursing.*

(13) I am anxious to return to India, after an absence of four years, and my friends there wish me to apply for a post as plague nurse. Can you tell me if plague nurses are still wanted, and to whom I should apply?—*M. H.*

Apply for full particulars to the Under Secretary, the India Office, St. James's Park, S.W.

*Hospital Training.*

(14) I am 21, and very anxious to become a hospital nurse. Do you think that I should be able to obtain a vacancy as probationer in a London hospital with a salary for the first year?—*G. H.*

You are too young for general nursing. You might be accepted however in a children's hospital.

I wish to enter the nursing profession. Is it best to enter a London hospital, and, if so, which one would you recommend?—*S. H. G.*

Consult "The Nursing Profession: How and Where to Train."

*Address.*

(15) Will you give me the address of the London Nursing Society.—*E. C.*

Do you mean the London Association of Nurses, 123 New Bond Street, W.?

*Opening.*

(16) I should be glad if you could tell me if there is a good opening for a maternity nurse in Clifton or Bristol.—*Shamrock.*

This is a matter on which we can offer no opinion. You should make inquiries locally.

---

**Standard Nursing Manuals.**

"The Nursing Profession: How and Where to Train." 2s. net; 2s. 4d. post free.

"Nursing: Its Theory and Practice." (Revised Edition). 3s. 6d. post free.

"Surgical Ward Work and Nursing." (Revised Edition). 3s. 6d. net.; 3s. 10d. post free.

"Practical Guide to Surgical Bandaging and Dressings." By Wm. Johnson Smith, F.R.C.S. 2s. post free.

"Practical Handbook of Midwifery." (New Edition). 6s. net; 6s. 3d. post free.

"Notes on Pharmacy and Dispensing for Nurses." 1s. post free.

"Fevers and Infectious Diseases." 1s. post free.

"The Art of Massage." (New Edition). 6s. post free.

### For Reading to the Sick.

---

"LORD, TO WHOM SHALL WE GO?"

When wounded sore the stricken heart
Lies bleeding and unbound,
One only Hand, a pierced Hand,
Can salve the sinner's wound.

When sorrow swells the laden breast,
And tears of anguish flow,
One only Heart, a broken Heart,
Can feel the sinner's woe.

When penitential grief has wept
Over some foul dark spot,
One only Stream, a Stream of Blood,
Can wash away the blot.

'Tis Jesus' Blood that washes white,
His Hand that brings relief,
His Heart is touch'd with all our joys,
And feels for all our grief.

Lift up Thy bleeding Hand, O Lord
Unseal that cleansing Tide:
We have no shelter from our sin
But in Thy wounded Side.

*Alexander.*

---

Our lives in this world are commonly likened to a pilgrimage or journey. We are ever hurrying on to an unknown land called eternity, and are unable to retrace a single step or go back over the ground once covered.

We look back from time to time and consider the past; and the vision of the past is apt to depress and discourage us, whereas, did we look at it aright, it should fill us with hope and gratitude and confidence in God.

We think of our many sins and shortcomings, of the graces and time we have wasted, of the awful difference between what we are and what we might be. All this is true, but it should only stimulate us to greater zeal and more earnest efforts for the future.

Let the sick apply this to themselves. Why did not God leave you as once you were, strong and vigorous, knowing no care, full of the joys of youth, with a life of wholesome enjoyment before you? You were doing no harm, you meant to say your prayers and keep near to God. How nice it would have been had all gone on as in those golden days! Why has a change come? Why have you been called to the sick-room, to the gate of death perhaps, to a bed of pain, to the active soldier-service of keen suffering and hardship? You know not the answer; but can you trust God to have done what was best for you? Can you trust Him to send you the greater graces which your present state requires? Are you ready to be an instrument in His hands for His work, or would you rather choose for yourself? We must avoid all worry, not thinking of the past in detail, but taking it as one great whole, in which we have had business to do for God and with God, in which God has been more than kind and generous, and over the failures of which He throws the mantle of His mercy and full forgiveness, for each sin has been dipped in the ocean of the Precious Blood and the name of Jesus is written across our account.—*Anon.*

---

We need as much the cross we bear
As air we breathe, as light we see,
It draws us to Thy side in prayer,
It binds us in our strength to Thee.

*A. L. Waring.*

## Figures and Tables

**Fig. 1. f1:**
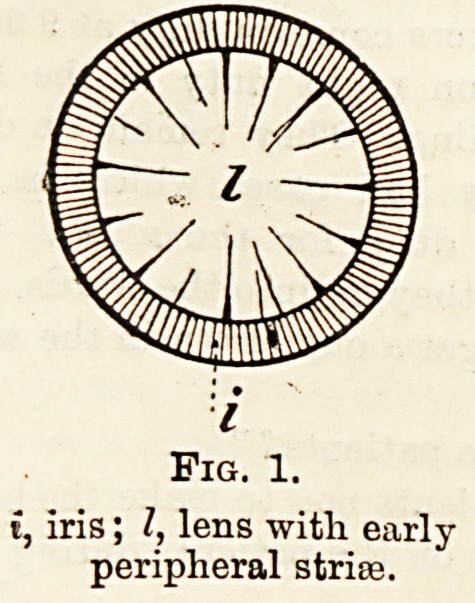


**Fig. 2. f2:**
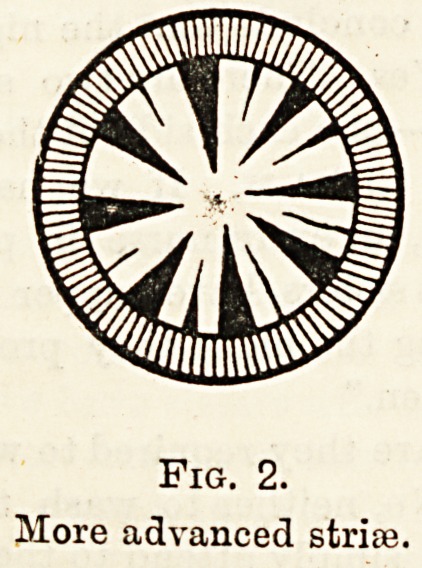


**Fig. 3. f3:**
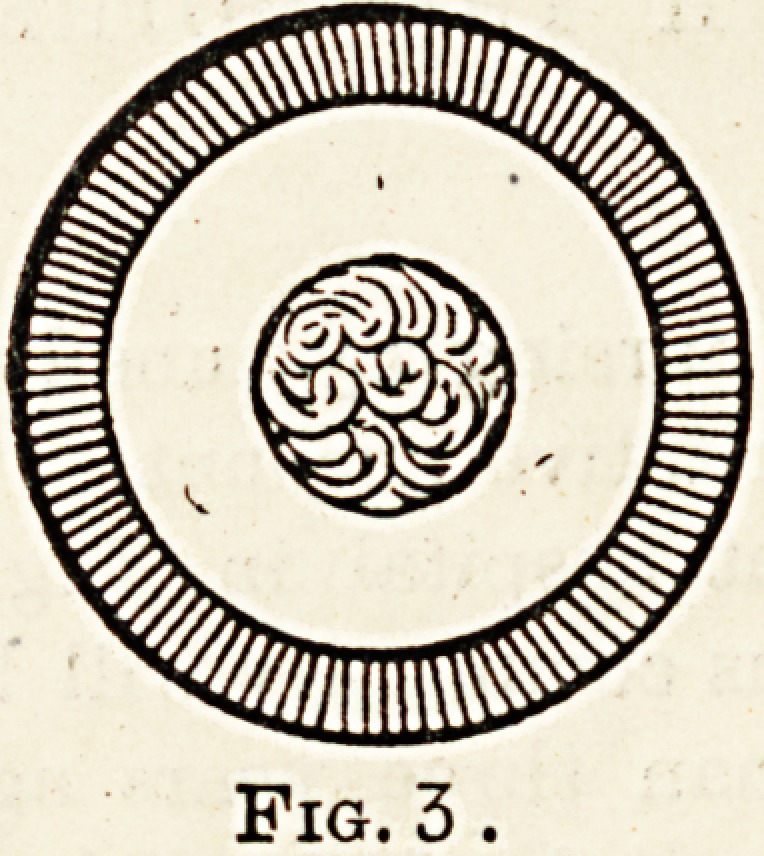


**Fig. 4. f4:**
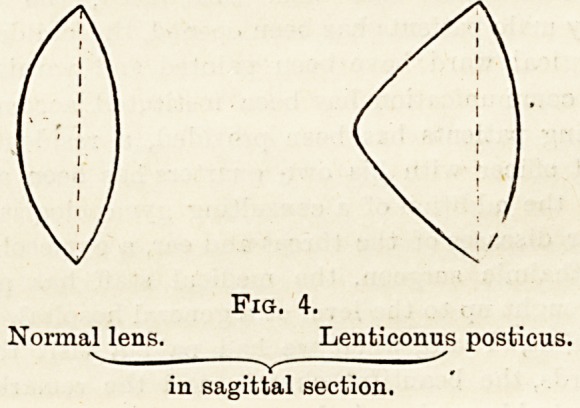


**Fig. 5. f5:**
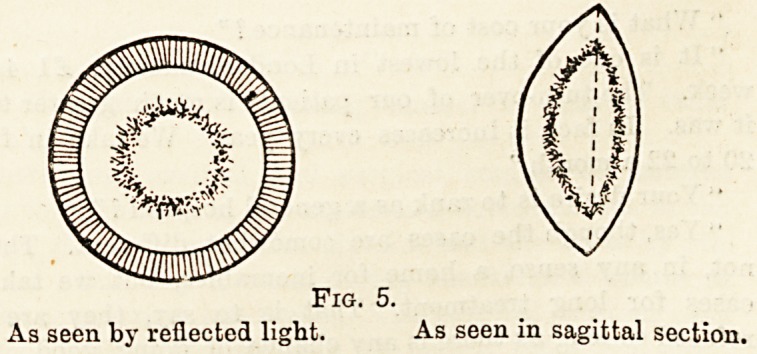


**Figure f6:**
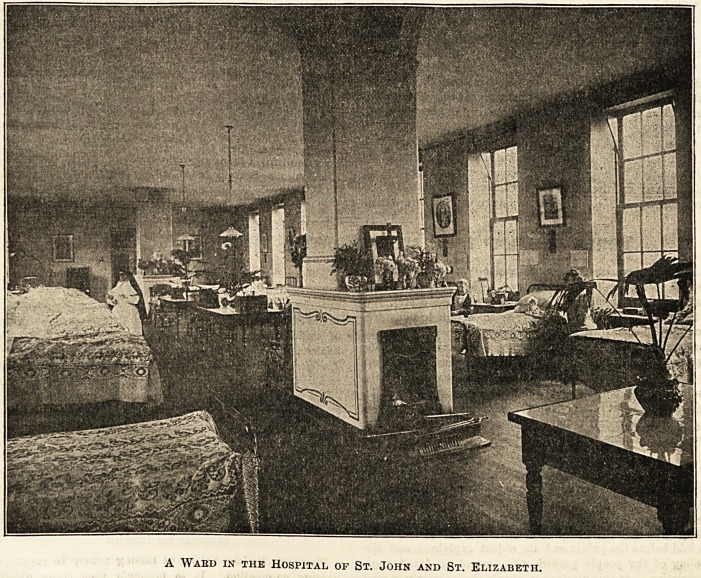


**Figure f7:**
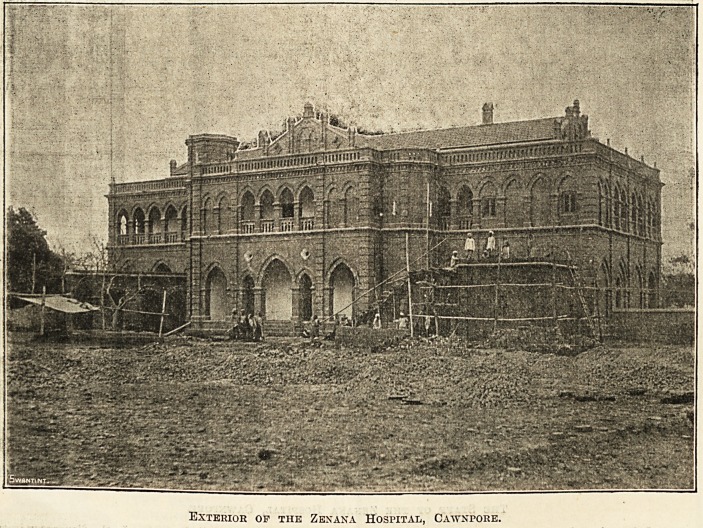


**Figure f8:**